# Dietary dicarboxylic acids provide a nonstorable alternative fat source that protects mice against obesity

**DOI:** 10.1172/JCI174186

**Published:** 2024-04-30

**Authors:** Eric S. Goetzman, Bob B. Zhang, Yuxun Zhang, Sivakama S. Bharathi, Joanna Bons, Jacob Rose, Samah Shah, Keaton J. Solo, Alexandra V. Schmidt, Adam C. Richert, Steven J. Mullett, Stacy L. Gelhaus, Krithika S. Rao, Sruti S. Shiva, Katherine E. Pfister, Anne Silva Barbosa, Sunder Sims-Lucas, Steven F. Dobrowolski, Birgit Schilling

**Affiliations:** 1 Department of Pediatrics, University of Pittsburgh School of Medicine, Pittsburgh, Pennsylvania, USA.; 2 The Buck Institute for Research on Aging, Novato, California, USA.; 3 Department of Pharmacology and Chemical Biology, University of Pittsburgh School of Medicine, Pittsburgh, Pennsylvania, USA.; 4 Health Sciences Mass Spectrometry Core, University of Pittsburgh, Pittsburgh, Pennsylvania, USA.; 5 Vascular Medicine Institute and; 6 Department of Pathology, University of Pittsburgh School of Medicine, Pittsburgh, Pennsylvania, USA.

**Keywords:** Metabolism, Fatty acid oxidation, Mitochondria, Obesity

## Abstract

Dicarboxylic fatty acids are generated in the liver and kidney in a minor pathway called fatty acid ω-oxidation. The effects of consuming dicarboxylic fatty acids as an alternative source of dietary fat have not been explored. Here, we fed dodecanedioic acid, a 12-carbon dicarboxylic (DC_12_), to mice at 20% of daily caloric intake for 9 weeks. DC_12_ increased metabolic rate, reduced body fat, reduced liver fat, and improved glucose tolerance. We observed DC_12_-specific breakdown products in liver, kidney, muscle, heart, and brain, indicating that oral DC_12_ escaped first-pass liver metabolism and was utilized by many tissues. In tissues expressing the “a” isoform of acyl-CoA oxidase-1 (ACOX1), a key peroxisomal fatty acid oxidation enzyme, DC_12_ was chain shortened to the TCA cycle intermediate succinyl-CoA. In tissues with low peroxisomal fatty acid oxidation capacity, DC_12_ was oxidized by mitochondria. In vitro, DC_12_ was catabolized even by adipose tissue and was not stored intracellularly. We conclude that DC_12_ and other dicarboxylic acids may be useful for combatting obesity and for treating metabolic disorders.

## Abstract 2

Dicarboxylic acids serve as an alternative energy source that dramatically remodels metabolism in mice.

## Graphical abstract

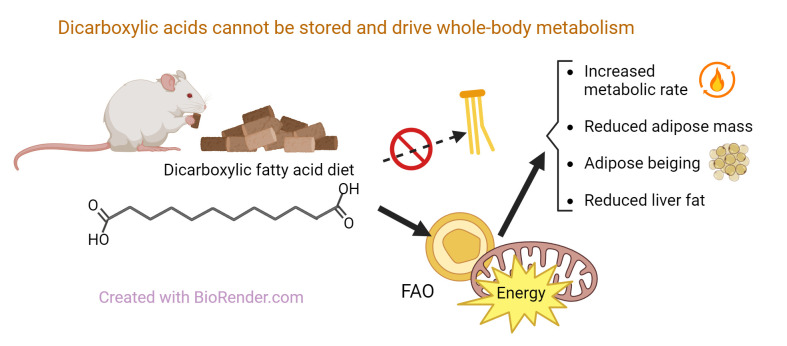

## Introduction

Changes in energy metabolism and mitochondrial function are noted across the spectrum of human diseases. First, there are numerous inborn errors of metabolism where chronic energy deficits and the risk of metabolic decompensation are primary to the disease. Second, common conditions such as obesity, type 2 diabetes, heart disease, chronic kidney disease, and Parkinson’s disease are underscored by declining bioenergetic capacity. However, because layers of regulatory mechanisms govern cellular uptake and oxidation of the 3 major metabolic substrates—glucose, fatty acids, and amino acids—therapeutically boosting energy metabolism is challenging. As a result, decades of research has sought to develop alternative substrates to replete energy stores while concurrently bypassing the molecular regulatory mechanisms that limit metabolic flux. 1 widely used alternative energy source is medium-chain triglycerides (MCT) (1). Oral MCT is hydrolyzed in the gut to free medium-chain fatty acids (MCFA), which can bypass the regulatory controls over fatty acid oxidation (FAO) due to their ability to cross membranes without facilitated transport. However, the therapeutic use of MCT has been limited, most likely due to strong first-pass liver metabolism. Because MCFA released in the gut cannot be reesterified and packaged into chylomicrons like long-chain fatty acids, they do not enter general circulation but, rather, are delivered in portal blood to the liver (2). There, MCFA undergo 3 fates: (a) oxidation for energy production and ketogenesis through the mitochondrial FAO pathway; (b) elongation to long-chain fatty acids followed by esterification and storage; and (c) conversion to dicarboxylic MCFA via a minor pathway dubbed ω-oxidation.

Fatty acids contain a carboxyl group at the α-carbon. The carboxyl moiety accepts the coenzyme-A (CoA) that is required for fatty acids to be biologically active, and the fatty acid is subsequently β-oxidized from this end. During MCFA ω-oxidation, a carboxyl group is added to the other “free” end (ω-carbon) of the fatty acid. This process occurs only in liver and kidney, and at appreciable levels only with C_10_ and C_12_ MCFA, although certain disease states or enzyme deficiencies may drive other substrates through this pathway (3). It begins with a hydroxylation step catalyzed by cytochrome P450 enzymes in the endoplasmic reticulum followed by 2 oxidation steps thought to occur in the cytosol (4). The resulting dicarboxylic acid (DCA) can be activated to CoA at either end and is preferentially β-oxidized by the peroxisomal FAO pathway, at least in the liver where the pathway has been best studied (4). The prevailing assumption regarding the biological role of ω-oxidation is that it serves to augment the disposal of excess fatty acids when the mitochondrial FAO pathway is saturated. The presence of MCFA in the cytosol would serve as a warning that partially chain-shortened intermediates are leaking out of mitochondria, and ω-oxidation followed by peroxisomal β-oxidation may be a fail-safe mechanism to remove these MCFA. In support of this theory, patients with genetic defects in mitochondrial medium-chain FAO — who accumulate medium-chain fatty acids — excrete large amounts of partially catabolized DCAs (DC_6_, DC_8,_ DC_10_) in urine, as do many people taking MCT supplements (5, 6).

We hypothesized that exogenous DCAs may be an attractive alternative energy source for several reasons. First and foremost, single-dose DCA test meals have been given to human subjects and were found to be safe (7–9); dodecanedioic acid and sebacic acid (hereafter DC_12_ and DC_10_, respectively) were both rapidly metabolized and improved exercise performance. Second, preferential oxidation of dietary fat by peroxisomal FAO would use less oxygen than the equivalent mitochondrial FAO pathway while also bypassing the strict regulatory control steps present in the mitochondrial pathway (i.e., carnitine palmitoyltransferase-1). Third, ex vivo experiments have shown that liver and kidney may chain-shorten DC_12_-CoA as far as DC_4_-CoA, better known as succinyl-CoA, which would be anaplerotic for the TCA cycle (10). Moreover, this succinyl-CoA might be released to circulation as succinate, which has recently been shown to remodel metabolic pathways in key tissues such as white adipose tissue (WAT) and muscle via its actions on the succinate receptor GRP91 (11, 12). Because very little is known about the metabolic effects of chronic DCA consumption, here we set out to characterize long-term DC_12_ feeding in mice.

## Results

### A high-fat diet substituted with DC_12_ increases metabolic rate and prevents obesity.

**Figure 1:**
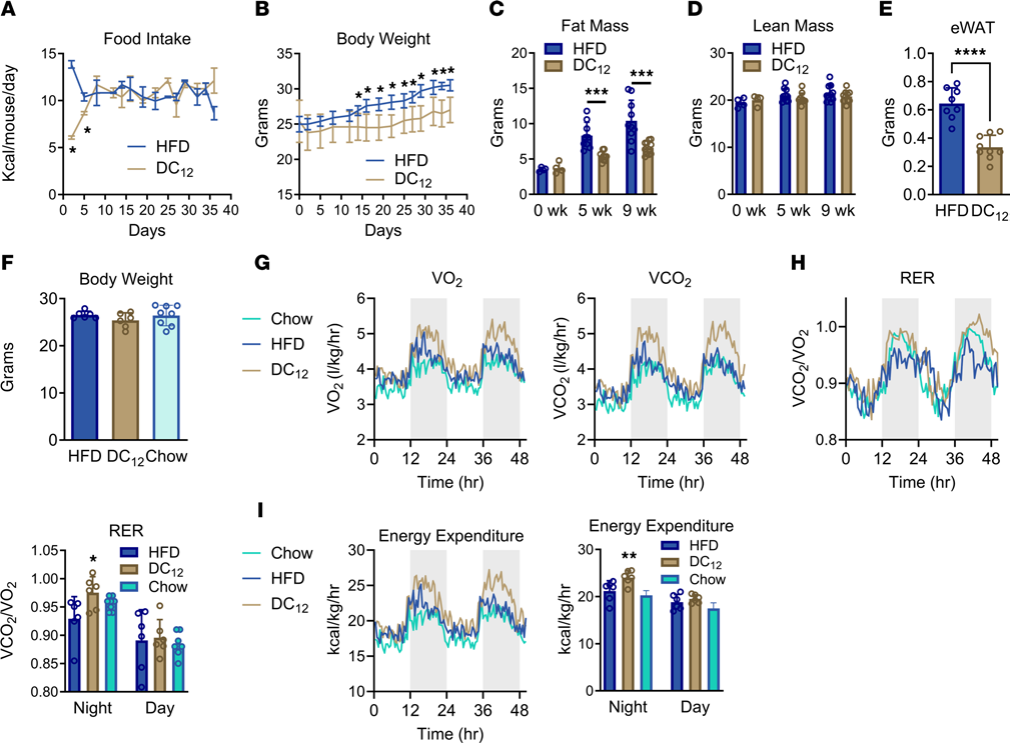
A HFD substituted with DC_12_ increases metabolic rate and prevents obesity. (**A** and **B**) Male 129S1 mice (*n* = 6) were transitioned to a HFD or the isocaloric DC_12_ diet at age 8 weeks. Food pellets were weighed every 2–3 days for 5 weeks to determine intake, and body weights were recorded every 2–3 days. (**C** and **D**) EchoMRI was used to assess total fat mass and lean mass after 5 weeks or 9 weeks of the special diets (*n* = 10). (**E**) Epididymal WAT (eWAT) was excised and weighed after 9 weeks on the diets. (**F**–**I**) HFD, DC_12_, and chow-fed control mice (*n* = 7–8) were subjected to indirect calorimetry after 7 days on the diets. Body weight was equal at the start of indirect calorimetry (**F**). Whole-body respiration was measured every 30 min over a 48 hr period (**G**). Panels **H** and **I** are the RER and energy expenditure calculated from the data in panel **G**, with each separated into night versus day cycles for statistical analysis. All graphs depict means and SDs. *P < 0.05, **P < 0.01, ***P < 0.001, ****P < 0.0001, by Student’s 2-sided *t* tests.

To interrogate the metabolic effects of exogenous DCA consumption, male 129S1 mice were transitioned to either a high-fat diet (HFD; 33% calories from soybean oil) or an isocaloric diet containing 10% w/w DC_12_ (21% calories from DC_12_, 12% from soybean oil; see Table 1 for diet composition). After initial adaptation to the DC_12_ diet, food consumption stabilized and was similar between groups (Figure 1A). By day 14, body weights had significantly diverged between the groups, with DC_12_-fed mice exhibiting progressively lower body weights than HFD mice (Figure 1B). EchoMRI was used to assess total body composition after 5 weeks and 9 weeks on the diets. At both time points, fat mass was significantly reduced in the DC_12_ group while lean mass was not changed (Figure 1, C and D). As a further indicator of reduced fat mass, the epididymal fat pad was excised and weighed. This fat depot was reduced approximately 50% in DC_12_-fed mice (Figure 1E). The experiment was repeated in the more obesity-prone C57BL/6J strain, which also showed reduced fat mass after consuming DC_12_ (Supplemental Figure 1, A and B; supplemental material available online with this article; https://doi.org/10.1172/JCI174186DS1). In female C57BL/6J mice, which are resistant to HFD-induced obesity compared with males (13), the effect of the diets on body weight and adiposity was blunted (Supplemental Figure 1, C–F). However, DC_12_ did reduce fat mass at the 9-week time point in females (Supplemental Figure 1F). Finally, male 129S1 mice were challenged with an ultra-high fat diet containing 60% of calories from long-chain fat versus an isocaloric diet containing 10% w/w DC_12_ (43% calories from long-chain fat, 17% calories from DC_12_; see Table 1 for full composition). Even in the context of ultra-high fat feeding, DC_12_ was able to significantly reduce body weight and fat mass while lean mass was unaltered (Supplemental Figure 1, G–J).

The reduced fat mass despite similar daily caloric intake suggested that DC_12_ may alter metabolic rate. To test this, mice were placed on the HFD or DC_12_ diets for 7 days and then subjected to indirect calorimetry. The 7-day time point was chosen to ensure that body weight had not yet diverged in the 2 groups, as body mass is a known confounding variable for rodents in indirect calorimetry (14). We also compared the 2 lipid-enriched diets to standard laboratory chow (hereafter, “chow”). At the time of indirect calorimetry, all 3 groups had similar body weights (Figure 1F). The rates of whole-animal O_2_ consumption and CO_2_ exhalation were both elevated in DC_12_-fed mice compared with HFD and chow mice (Figure 1G). The ratio of VCO_2_/VO_2_, known as the respiratory exchange ratio (RER), is considered an indicator of the substrate being oxidized, because fat oxidation consumes more oxygen per CO_2_ exhaled than carbohydrate oxidation. Theoretical RER values range from 0.7 CO_2_ exhaled per O_2_ consumed, which represents a complete reliance on mitochondrial fat oxidation, to 1.0, which represents a complete reliance upon glucose oxidation. Here, during the dark cycle while mice are actively feeding, we observed the RER in DC_12_-fed mice to be similar to that of chow-fed mice and significantly higher than that of HFD-fed mice (Figure 1H). During the light cycle, while mice are mostly resting, all 3 groups displayed similar RERs. Likewise, the calculated energy expenditure in DC_12_-fed mice was significantly higher, but again only during the dark cycle (Figure 1I). The increase in metabolic rate caused by chronic DC_12_ consumption likely is the cause of the reduced accrual of fat mass over time. After 5 weeks on the diets, the divergence in body mass rendered it difficult to accurately compare metabolic rate across the HFD and DC_12_ groups. However, we did calculate RER, a ratio which is independent of body mass. Again, DC_12_ consumption was associated with a higher nighttime RER, but not daytime RER, compared with HFD (Supplemental Figure 1K).

### DC_12_-fed mice remain glucose-sensitive.

**Figure 2:**
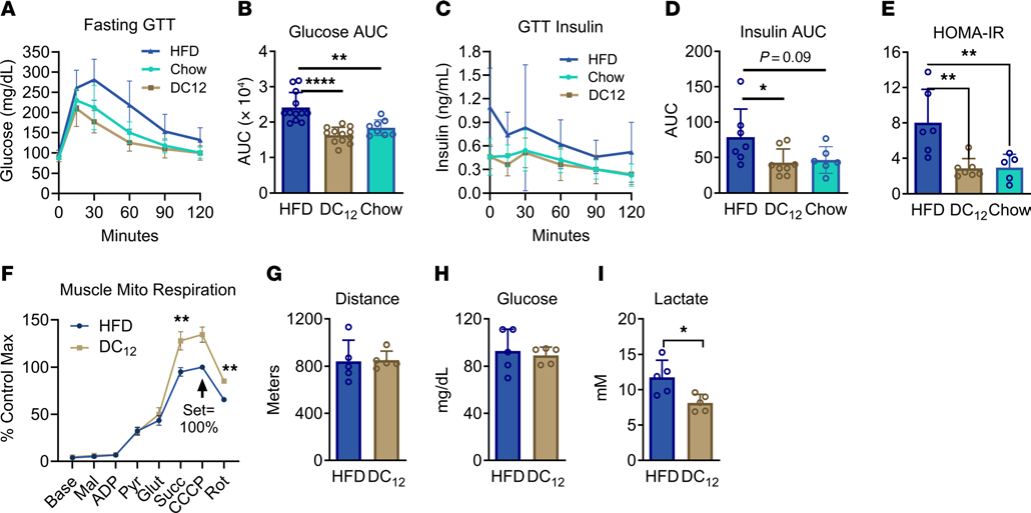
DC_12_-fed mice remain glucose-sensitive. (**A**–**D**) Male 129S1 mice (*n* = 8–12) were fed a HFD, the isocaloric DC_12_ diet, or standard chow for 5 weeks and then subjected to i.p. glucose tolerance testing (GTT) after a 5-hr fast. (**E**) Blood glucose and insulin data at baseline (time 0 of the GTT) was used to calculate homeostatic model assessment (HOMA) values as an indicator of insulin sensitivity. (**F**) Oroboros high-resolution respirometry of quadriceps muscle lysates (*n* = 3). Base, baseline; mal, malate; pyr, pyruvate; glut, glutamate; succ, succinate; CCCP, mitochondrial uncoupler; rot, rotenone. (**G**–**I**) Acute treadmill exercise challenge to exhaustion (*n* = 5). Blood lactate and glucose were measured with handheld meters within 2 minutes of reaching exhaustion. All graphs represent means and SDs. Panels **B**, **D**, and **E** were analyzed with 1-way ANOVA and Tukey-corrected multiple comparisons and remaining panels were analyzed with 2-sided Student’s *t* test. *P < 0.05, **P < 0.01, ****P < 0.0001.

Increased fat mass is well known to be associated with a syndrome of metabolic dysfunction that includes glucose intolerance and reduced mitochondrial function. Compared with HFD, DC_12_-fed mice exhibited protection against these impairments. Glucose tolerance testing was performed during the light cycle after a 5-hour fast in mice that had been maintained on HFD, DC_12_, or standard chow diet for 5 weeks. The DC_12_-fed animals cleared the intraperitoneal glucose bolus at a similar rate as chow-fed controls, while clearance was impaired in HFD mice (Figure 2, A and B). Serum insulin levels were also significantly higher in HFD mice compared with DC_12_-fed mice (Figure 2, C and D). Homeostatic model assessment for insulin resistance (HOMA-IR) values, calculated from the time 0 glucose and insulin concentrations as an indicator of insulin resistance, were significantly higher in HFD mice (Figure 2E). We then assessed glucose tolerance at night, in the fed-state, in HFD and DC_12_-fed mice of both sexes. In males, fed-state glucose tolerance was impaired in HFD mice compared with DC_12_-fed (Supplemental Figure 2A). In female mice the effect trended in the same direction but was not significant, likely due to their general resistance to the effects of HFD (Supplemental Figure 2B). Finally, 2 recent studies showed that HFD leads to reduced muscle mitochondrial respiratory capacity over time (15, 16). Here, muscle mitochondria isolated from DC_12_-fed animals respired more robustly than those from HFD mice (Figure 2F). Liver mitochondrial respiration did not significantly differ between the 2 groups (Supplemental Figure 2C). Because of declining muscle mitochondrial efficiency, HFD-fed mice have been shown to ramp up glycolytic activity in muscle during exercise, leading to a more rapid accumulation of lactate in blood (17). HFD and DC_12_-fed mice were subjected to an acute run-to-exhaustion challenge on a treadmill. The 2 groups ran similar distance and had similar blood glucose values at exhaustion, but the DC_12_-fed mice had approximately 40% lower blood lactate values at exhaustion (Figure 2, G–I).

### Dietary DC_12_ is excreted only in trace amounts and is not stored intracellularly.

**Figure 3:**
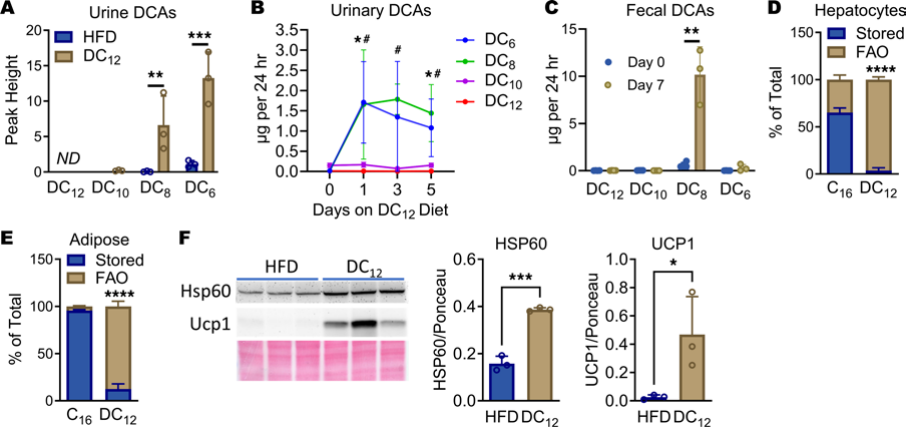
Dietary DC_12_ is excreted only in trace amounts and is not stored intracellularly. (**A**) Male 129S1 mice (*n* = 3) were adapted to either a HFD or an isocaloric DC_12_ diet for 5 weeks, and nighttime urine was collected for mass spectrometry to detect DCAs. (**B**) Male 129S1 mice (*n* = 4) were adapted to the DC_12_ diet over a 5 day period, with 24-hr urine samples collected on days 0, 1, 3, and 5 for mass spectrometry to detect DCAs. (**C**) Similarly, fecal pellets were collected from *n* = 3–4 male 129S1 mice on days 0 and 7 of DC_12_ adaptation for mass spectrometry. (**D** and **E**) Primary hepatocytes (**D**) or white adipose explants (**E**) were incubated with ^14^C-labeled palmitate (C_16_) or DC_12_ for 3 hr, washed, and extracted for lipids and FAO products (*n* = 5).The amount of stored versus oxidized are expressed as a percentage of the total radiolabel signal detected. (**F**) WAT (*n* = 3) blotted for the mitochondrial marker Hsp60 and Ucp1; bar graphs show densitometric analysis normalized to ponceau stain. All graphs represent means and SDs. In panel **B**, *P < 0.05, DC_6_ versus Day 0; ^#^*P* < 0.05, DC_8_ versus Day 0. In remaining panels: **P < 0.01, ***P < 0.001, ****P < 0.0001. All were analyzed with 2-sided Student’s *t* test.

Previous studies dosed rats and humans with single oral boluses of DC_12_ and observed approximately 3% of the dose (or less) was excreted in urine (18–20). Excretion has not been studied in the context of chronic dietary consumption. If our DC_12_-fed mice were excreting a substantial amount of DC_12_ carbon, this loss of unmetabolized calories could contribute to the attenuated accrual of fat mass over time. To interrogate this issue, we began by collecting 12-hour (dark cycle) urine samples from mice fed DC_12_ for 5 weeks and measuring urinary DCAs by gas chromatography mass spectrometry. We focused on DC_12_ and shorter DCAs that could be unequivocally assigned as chain-shortened products of DC_12_ (DC_10_, DC_8_, and DC_6_). DC_12_ itself was not detected in urine from either group. DC_10_ was also not detected in HFD-fed mice but was present in trace amounts in DC_12_-fed mice. The most abundant DCA was DC_6_ (adipate), a well-known marker for the fatty acid ω-oxidation pathway (5, 21), which was increased approximately 13-fold in DC_12_-fed mice (Figure 3A). DC_8_ was also significantly increased in urine from DC_12_-fed mice.

To more accurately quantify the amount of DC_12_-based carbon being excreted, we followed a cohort of 129S1 mice for 5 days as they adapted to the DC_12_ diet. Urine was collected from singly housed animals at 24 hour intervals and the volume recorded. Food intake was recorded. The concentration of DCAs was then determined by a more sensitive, targeted liquid chromatography mass spectrometry (LC-MS) assay and multiplied by urine volume to yield absolute amounts (in μg) excreted during each 24 hour period. With this more sensitive assay, DC_12_, DC_10_, DC_8_, and DC_6_ were all above the limit of detection even at baseline. For DC_12_ and DC_10_, the detected amounts were very low, in nanogram amounts, and did not change significantly upon consumption of exogenous DC_12_ (Figure 3B). DC_8_ and DC_6_ both rose dramatically and somewhat variably (approximately 100-fold) during the first 24 hours after switching to the DC_12_ diet, and then declined slightly out to Day 5. Next, we collected and weighed feces over 24 hour periods in a cohort of mice at baseline (Day 0) and on Day 7, and subjected fecal pellets to our LC-MS assay. As with urine, all 4 DCAs were above the limit of detection. Interestingly, in feces, only DC_8_ was significantly increased by DC_12_ feeding (Figure 3C). Fecal metabolites are thought to reflect the metabolism of the microbiome, which would not normally encounter DCAs as they are not present in any common food source. This finding may reflect an incapacity of the microbiome to chain-shorten dietary DCAs past 8 carbons. Most importantly, this urine and fecal data, when combined with recorded food intake for each mouse, allowed us to estimate that approximately 0.03% of consumed DC_12_ carbon is excreted in urine as partially shortened products DC_8_ and DC_6_, and another approximately 0.07% in feces as DC_8_. Therefore, the amount of dietary DC_12_ calories lost by excretion is inconsequential.

Based on these low excretion rates, coupled with increased energy expenditure in the dark cycle when DC_12_ is being consumed, we hypothesized that DC_12_ is completely oxidized as it is consumed. In other words, during the dark cycle, when insulin is high and dietary fat is normally directed preferentially to storage, mice on the DC_12_ diet are actively engaged in FAO. However, the RER of DC_12_-fed mice was similar to chow-fed control and did not reflect a higher rate of FAO, which was at odds with our hypothesis. We therefore set out to experimentally test whether DC_12_ could be stored intracellularly. To do this, we employed ^14^C-labeled DC_12_ and followed partitioning to either FAO products or intracellular storage. First, primary mouse hepatocytes were incubated with either ^14^C-DC_12_ or ^14^C-C_16_ (as a control fatty acid) in the presence of insulin to promote storage. After 3 hours the cells were washed and subjected to chloroform-methanol extraction. For DC_12_, the radiolabel was nearly all in the aqueous layer (FAO products) and no DC_12_ was stored intracellularly (Figure 3D). In striking contrast, more than 60% of ^14^C-C_16_ taken up under these conditions was stored. In addition to liver, WAT is also a major tissue site for dietary fatty acid uptake and storage. WAT explants incubated with ^14^C-DC_12_ contained predominantly oxidation products, and very little DC_12_ was detected within the tissue lipid fraction. Again, in striking contrast, nearly all the ^14^C-C_16_ that was taken up by WAT explants was stored intracellularly (Figure 3E). We also saw additional in vivo evidence that the DC_12_ diet remodels metabolism in WAT. WAT from mice on DC_12_ for 9 weeks exhibited smaller adipocyte size (Supplemental Figure 3A). Further, DC_12_ induced increased expression of heat shock protein-60 (HSP60), a marker of mitochondrial abundance, and uncoupling protein-1 (UCP1) in WAT (Figure 3F). This suggests that uptake and oxidation of DC_12_ within adipocytes may induce “beiging” of WAT. This finding led us to further interrogate brown adipose tissue (BAT), the major thermogenic tissue in rodents. BAT from DC_12_-fed mice had smaller intracellular lipid droplets (Supplemental Figure 3B). However, there was no change in total BAT weight, UCP1 expression, or HSP60 expression, and further, no difference was seen in core body temperature of DC_12_-fed males or females when measured at night with a rectal probe (Supplemental Figure 3, C–F).

### DC_12_ is metabolized using both peroxisomes and mitochondria.

**Figure 4:**
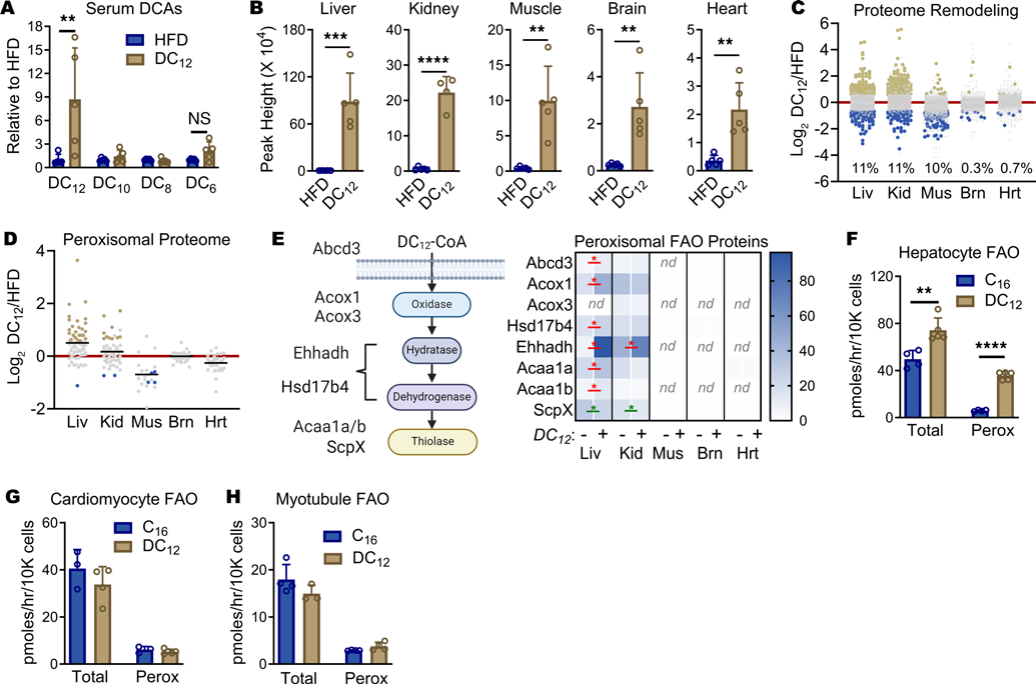
DC_12_ is metabolized using both peroxisomes and mitochondria. (**A** and **B**) Male 129S1 mice (*n* = 5) were adapted to HFD or an isocaloric DC_12_ diet for 5 weeks. Serum was collected early during the night cycle, and tissues late in the night cycle, and they were used for mass spectrometry to detect DCAs. (**C** and **D**) Proteomics results from tissues collected after 5 weeks on the diets (*n* = 3), expressed as log_2_ of the fold-change of DC_12_-treated animals over HFD. Also see proteomics data in Supplemental Tables 1–6. Gold dots represent proteins that were significantly increased and blue dots are proteins that were significantly decreased (absolute log_2_FC > 0.58, *q* < 0.05), while gray are proteins with no significant change. Black lines indicate the mean of each condition. (**E**) Heatmap showing the absolute levels of key peroxisomal FAO proteins across the 5 different tissues. Heatmap values are means of *n* = 3; ND, not detected. Asterisks indicate statistically significant pairwise differences (*q* < 0.01), either upregulated by DC_12_ diet (red font) or downregulated (green font). (**F**–**H**), ^14^C-labeled DC_12_ or palmitate (C_16_) were used to probe the rates of total FAO (no etomoxir) or peroxisomal FAO (etomoxir-resistant) in whole cells. All bar graphs represent means and SDs. *P < 0.05, **P < 0.01, ***P < 0.001, ****P < 0.0001 as determined with 2-sided Student’s *t* test. Liv, liver; Kid, kidney; Mus, muscle; Brn, brain; and Hrt, heart. Panel **E** created with BioRender.com.

Fatty acid ω-oxidation, which produces endogenous DCAs, occurs strictly in the liver and kidney. What is known about the catabolism of DCAs comes almost exclusively from the study of these 2 organs, particularly the liver. The consensus of the literature is that, in liver, DCAs are metabolized preferentially by peroxisomes, although the mitochondrial FAO pathway can assist in situations of peroxisomal dysfunction (22–25). Peripheral organs such as muscle, heart, and brain have little exposure to endogenous DCAs. It is not clear to what extent they are capable of DCA catabolism or by which pathway. We set out to explore these questions in mice consuming exogenous DC_12_. First, we collected serum from mice during the night while actively consuming their respective diets (HFD or DC_12_) and measured circulating DCAs. The concentration of serum DC_12_ was increased 9-fold in mice on the DC_12_ diet, confirming that oral DC_12_ can at least partially escape first-pass liver metabolism (Figure 4A). Unlike urine, where chain-shortened products like DC_6_ and DC_8_ predominated, in serum there was no significant change in these products, although DC_6_ exhibited a nonsignificant upward trend.

Next, we asked whether DC_12_-specific catabolic products could be found in extrahepatic tissues. Five highly metabolic organs—liver, brain, heart, muscle, and kidney—were harvested at the end of the dark cycle. Mass spectrometry identified the DC_12_ product DC_6_, but not DC_8_ or DC_10_, in all 5 tissues, and DC_6_ levels were significantly increased by DC_12_ feeding (Figure 4B). This indicated that many different tissues have the capacity to catabolize DCAs, but provided no information regarding whether it occurred through peroxisomal or mitochondrial FAO. We therefore subjected these same 5 organs to proteomics to gain insight into the relative abundance of the peroxisomal FAO machinery. DC_12_ significantly remodeled the proteome in liver, kidney, and muscle (Figure 4C and Supplemental Tables 1–6), which were the 3 tissues with the most robust DC_6_ signals in Figure 4B. Curating known peroxisomal proteins out of this data set, it was seen that the peroxisomal content of the 5 tissues varied widely, as did the response of the peroxisomal proteome to DC_12_ (Figure 4D). Peroxisomes are small organelles with under 100 proteins, believed to be present in nearly every cell type of the body (26). The total number of peroxisomal proteins we detected was 68 (liver), 57 (kidney), 38 (brain), 28 (heart), and 22 (muscle).

The peroxisomal enzymes ATP Binding Cassette Subfamily D Member 3 (ABCD3), acyl-CoA oxidase-1 (ACOX1), enoyl-CoA hydratase/3-hydroxy-acyl-CoA dehydrogenase (EHHADH), and 3-ketoacyl-CoA thiolase (ACAA1) have all been shown to play a role in peroxisomal DCA catabolism (25, 27–29). Note that there are 2 *Acaa1* genes in mouse, dubbed *Acaa1a* and *Acaa1b*. Additionally, 3 other peroxisomal FAO enzymes have partial functional overlap with the above proteins and may also contribute to DC_12_ oxidation: acyl-CoA oxidase-3 (ACOX3), hydroxysteroid 17-β dehydrogenase 4 (HSD17B4), and sterol carrier protein-X (SCPX) (4, 28, 30, 31). Notably, liver expressed 7 of these 8 peroxisomal FAO enzymes and kidney expressed all 8 (Figure 4E). Liver responded to DC_12_ feeding by significantly upregulating ABCD3, ACOX1, HSD17B4, EHHADH, ACAA1a, and ACAA1b, while in kidney only EHHADH was upregulated. In both tissues SCPX was modestly downregulated by DC_12_ (Figure 4E). In contrast to liver and kidney, muscle, brain, and heart expressed only a minimum complement of peroxisomal FAO enzymes, and at low levels (Figure 4E). Each expressed low levels of ACOX1, HSD17B4, ACAA1a, and SCPX, such that all 4 enzymatic steps of peroxisomal β-oxidation are represented. Immunoblotting confirmed the very low expression of ACOX1 in muscle, heart, and brain, and the absence of EHHADH in these tissues (Supplemental Figure 4, A and B).

The proteomics findings raised the question of whether peroxisomes contribute to the degradation of DC_12_ in tissues outside the liver and kidney. Mitochondria have previously been shown to chain-shorten DC_12_ and DC_10_, but other studies refute this (23, 24, 32). In pilot experiments with ^14^C-labeled DC_12_, we noted that both primary hepatocytes and primary cardiomyocytes were able to produce ^14^C-CO_2_ when incubated with ^14^C-DC_12_ (Supplemental Figure 4, C and D). However, peroxisomal FAO does not produce CO_2_. The observed ^14^C-CO_2_ could only arise by either the transfer of chain-shortened products from peroxisomes into mitochondria or by direct mitochondrial FAO of ^14^C-DC_12_. We therefore tested whether ^14^C-DC_12_ oxidation was sensitive to etomoxir, an irreversible inhibitor of the key mitochondrial FAO enzyme carnitine palmitoyltansferase-1 (CPT1), in primary hepatocytes, primary cardiomyocytes, and differentiated myotubules. ^14^C-palmitate (C_16_), a known mitochondrial FAO substrate, was evaluated as a positive control. In hepatocytes, ^14^C-DC_12_ was oxidized at a higher total rate than ^14^C- C_16_ (Figure 4F). The peroxisomal portion (etomoxir-resistant) was approximately 6 × higher for DC_12_ compared with C_16_. In contrast, both cardiomyocytes and myotubules oxidized DC_12_ in a manner that was indistinguishable from that of the mitochondrial substrate C_16_, exhibiting over 90% inhibition by etomoxir (Figure 4, G and H). We conclude that heart and muscle oxidize DC_12_ almost exclusively through mitochondrial FAO.

### Consumption of DC_12_ does not cause dyslipidemia or fatty liver.

**Figure 5:**
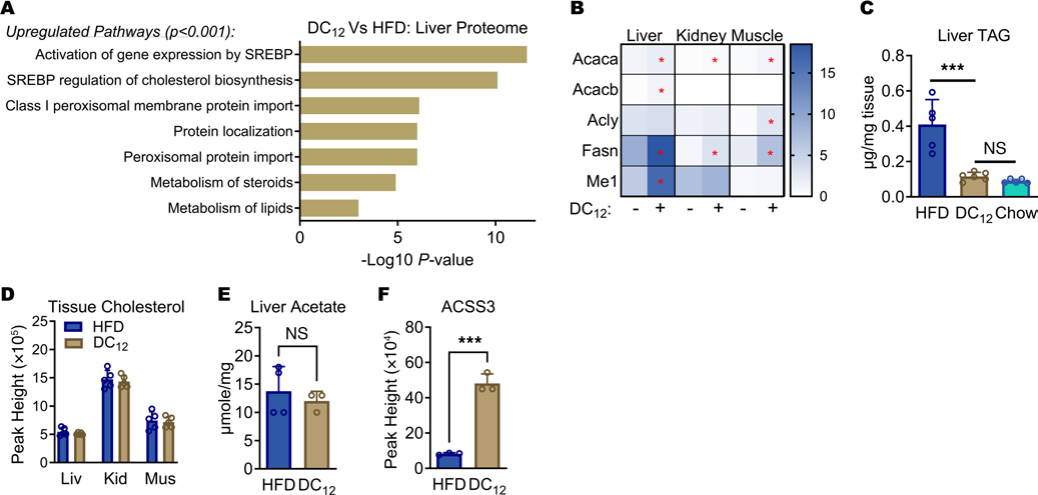
Consumption of DC_12_ does not cause dyslipidemia or fatty liver. (**A** and **B**) Reactome pathway analysis of the proteomics data presented in Figure 4 revealed an upregulation of lipid synthesis pathways in liver of DC_12_-fed mice versus HFD (**A**), and (**B)** fatty acid synthesis proteins were also upregulated in kidney and muscle. Red asterisks denote statistically significant upregulation in DC_12_ versus HFD. See also Supplemental Tables 1–6. (**C**) Liver triglyceride (TAG) content of 129S1 male mice on HFD or DC_12_ diet for 5 weeks compared with mice fed standard low-fat laboratory chow (*n* = 5–6). (**D**) Mass spectrometry was used to measure cholesterol content in mouse tissues (Liv; liver; Kid, kidney; Mus, muscle) after 5 weeks on special diets (*n* = 5). (**E**) Liver free acetate content (*n* = 3–4) was determined with a colorimetric kit. (**F**) Proteomics identified short-chain ACSS3 as being upregulated by DC_12_ in liver. All bar graphs represent means and SDs. *P < 0.05, **P < 0.01, ***P < 0.001, as determined with 2-sided Student’s *t* test.

The data presented above suggest that dietary DC_12_ constitutively stimulates peroxisomal FAO in liver. Previous studies have observed increased hepatic peroxisomal FAO in HFD-fed mice and linked this increase to fatty liver (33–35). It is generally accepted that acetyl-CoA produced within peroxisomes during fatty acid chain–shortening cannot cross the peroxisomal membrane and is therefore released either as free acetate or as acetylcarnitine (36). Reesterification of acetate to acetyl-CoA in the cytoplasm might induce lipogenesis or fatty acid chain elongation. We therefore investigated whether DC_12_ promotes lipogenesis and fatty liver. First, we performed pathway analysis on the deregulated proteins found in our proteomics survey described above (see Figure 4), which revealed a clear upregulation of cholesterol and fatty acid synthesis enzymes in DC_12_-fed liver (Figure 5A and Supplemental Figure 5A). The upregulation of fatty acid synthesis genes was also present in kidney and muscle, although to a lesser degree (Figure 5B). However, there was no indication of dyslipidemia in DC_12_-fed mice. HFD-fed mice had, on average, 4 times more liver triglyceride than chow-fed controls after 5 weeks on the diet, while DC_12_-fed mice remained similar to chow-fed animals (Figure 5C). Supplementation of a 60% ultra-HFD with DC_12_ also significantly reduced liver triglycerides (Supplemental Figure 5B). Tissue levels of saturated (C_16_) and unsaturated (C_18:1_) fatty acids were not altered by DC_12_ feeding in liver, kidney, or muscle, nor was cholesterol (Supplemental Figure 5, C and D, and Figure 5D). At night, while the animals were actively feeding, handheld meters were used to spot-check serum cholesterol and triglycerides. Both were reduced in DC_12_-fed mice (Supplemental Figure 5E).

Finally, we asked whether DC_12_ increased the amount of free acetate or acetylcarnitine due to a drive on peroxisomal FAO. In DC_12_-fed mice, acetate levels in liver and serum were not significantly different from HFD controls (Figure 5E and Supplemental Figure 5F). Unexpectedly, acetylcarnitine was significantly lower in serum collected from DC_12_-fed mice at night, not higher, as would be expected if liver peroxisomes were actively exporting acetylcarnitine, while daytime acetylcarnitine levels did not differ between diet groups (Supplemental Figure 5, G and H). Together, these findings suggest that DC_12_ consumption does not cause dyslipidemia, and, further, that in liver, the 2-carbon units produced by peroxisomal metabolism of DC_12_ are not being released to circulation. Interestingly, liver proteomics revealed that DC_12_ induced a 6-fold increase in the abundance of acetyl-CoA synthetase-3 (ACSS3), a recently identified mitochondrial matrix enzyme capable of activating free acetate to acetyl-CoA for oxidation by the TCA cycle (37) (Figure 5F). We speculate that DC_12_ may induce ACSS3 expression to dispose of acetate released by peroxisomes.

### Dietary DC_12_ is chain-shortened to succinyl-CoA in several tissues, but circulating succinate is not increased.

Ex vivo evidence suggests that the degradation of DC_12_ involves 4 cycles of chain shortening, producing 4 acetyl-CoA and a remnant DC_4_-CoA, which is better known as succinyl-CoA (10, 38). While succinyl-CoA cannot cross membranes unassisted, peroxisomes contain the enzyme acyl-CoA thioesterase-4 (ACOT4) which cleaves the CoA, yielding free succinate that readily leaves the peroxisome (39). Succinate has recently become recognized as a powerful signaling molecule that binds to the succinate receptor GPR91 on the cell surface of target tissues such as WAT and muscle to remodel metabolism within these tissues. Based on this emerging literature we hypothesized that dietary DC_12_ serves as a source of succinate and exerts its protective effects against obesity via succinate signaling.

**Figure 6:**
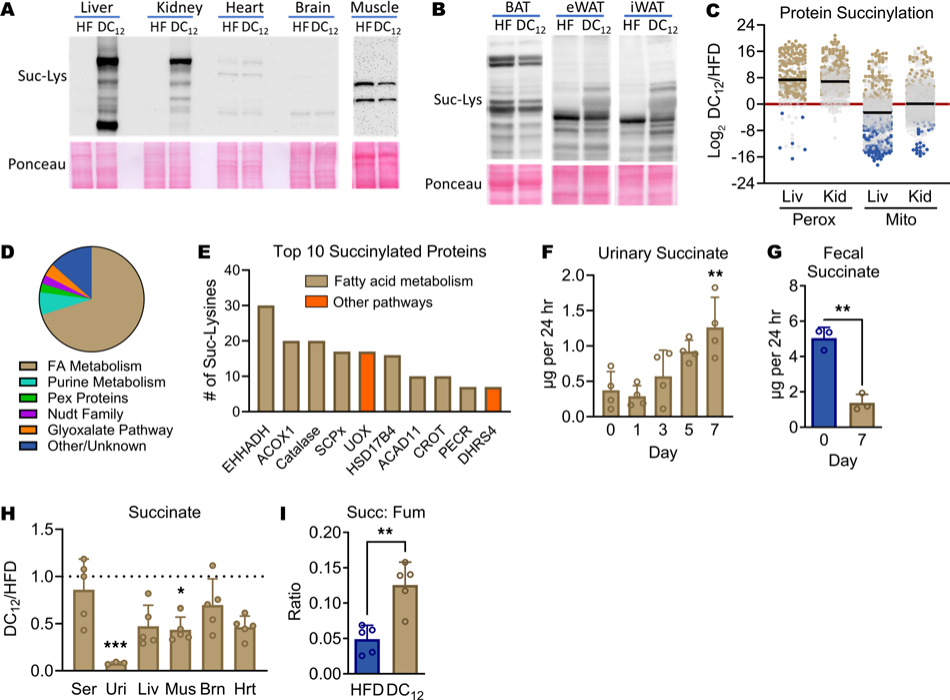
Dietary DC_12_ is chain shortened to succinyl-CoA in several tissues, but circulating succinate is not increased. (**A**) Immunoblotting of 20 μg of mouse tissue lysates after 7 days on HFD (HF) or DC_12_ diets with a pan anti-succinyllysine (Suc-Lys) antibody, with Ponceau staining as loading control. Note: To visualize muscle succinylation, 40 μg protein and a longer exposure time were needed. (**B**) Anti-succinyllysine immunoblotting of BAT, epididymal WAT (eWAT), and inguinal WAT (iWAT). (**C**) Liver and kidney extracts from mice on DC_12_ or HFD (*n* = 4) were used for quantitative site-level succinylomics by mass spectrometry. Peroxisomal and mitochondrial peptides were curated and plotted as log_2_ fold-change (DC_12_/HFD) to visualize the effects of DC_12_ on succinylation in each compartment. Gold dots represent peptides with significantly increased succinylation, blue dots represent peptides with significantly decreased succinylation, and gray indicates statistical insignificance. (**D**) Pathway analysis of all succinylated peroxisomal proteins in liver reveals strong clustering to the fatty acid metabolism pathway; the most heavily succinylated peroxisomal proteins are depicted in **E**. See Supplemental Tables 7 and 8 for succinylome data sets and full protein names. (**F** and **G**) Mass spectrometry was used to measure succinate in urine and feces from male 129S1 mice during the initial 7 days of adaptation to DC_12_ diet. (**H**) After chronic adaptation to the DC_12_ diet or HFD (5 wk), mass spectrometry was used to quantify succinate in serum, urine, liver, muscle, brain, and heart. Succinate is presented as a ratio of DC_12_: HFD, and the dashed line represents no change (ratio of 1.0). (**I**) The ratio of succinate to fumarate represents the substrate: product ratio for the enzyme succinate dehydrogenase, the entry point of succinate in the TCA cycle. Panel **F** was analyzed with 1-way ANOVA and Tukey-corrected multiple comparisons, and remaining panels were analyzed with 2-sided Student’s *t* test. *P < 0.05, **P < 0.01, ***P < 0.001. Ser, serum; Uri, urine; Liv, liver; Kid, kidney; Mus, muscle; Brn, brain; Hrt, heart.

Succinyl-CoA is highly labile (40). Because of its chemical instability, succinyl-CoA spontaneously forms succinic anhydrides that chemically react with proteins to produce the posttranslational modification known as lysine succinylation (41). In vivo, protein succinylation levels change with succinyl-CoA levels (42–44). We therefore used lysine succinylation as a proxy measure for succinyl-CoA formation in DC_12_-fed mice. We began by screening tissue lysates for protein succinylation using a pan anti-succinyllysine antibody. In mice consuming the DC_12_ diet, there was a marked increase in total protein succinylation in liver and kidney, but not brain, heart, or skeletal muscle (Figure 6A). In BAT we observed a slight decrease in the overall protein succinylation levels upon DC_12_ feeding, whereas succinylation was modestly increased in WAT (Figure 6B). This supports data presented in Figure 3, suggesting that DC_12_ promotes beiging of WAT but has little effect on BAT. We then chose liver and kidney, which exhibited the most robust change in protein succinylation, for further study. In both tissues, the level of protein succinylation took 5-to-7 days of DC_12_ feeding to reach maximal levels (Supplemental Figure 6). After 5 weeks of DC_12_ feeding, liver and kidney tissues were harvested for mass spectrometry to quantify changes to the lysine succinylome at the residue level (normalized to protein abundance). In both tissues, consumption of DC_12_ was associated with a dramatic increase in succinylation of peptides belonging to peroxisomal proteins (Figure 6C), with average increases of 170-fold and 120-fold in liver and kidney, respectively, compared with chow-fed controls (see also Supplemental Tables 7 and 8). In contrast, the abundance of succinylation on mitochondrial proteins tended to either decrease (liver) or stay the same (kidney). More than 70% of the succinylated peptides identified by mass spectrometry in liver peroxisomes belonged to enzymes involved in fatty acid metabolism and 8 of the top 10 most succinylated proteins were fatty acid metabolism enzymes, including enzymes involved in DC_12_ catabolism such as EHHADH, ACOX1, and catalase, which is the redox partner for ACOX1 (Figure 6, D and E).

The observed compartment-specific increase in protein succinylation in liver and kidney suggests that DC_12_ is chain shortened to succinyl-CoA primarily within peroxisomes of these tissues. Through the action of ACOT4, this succinyl-CoA can be released as free succinate, which could then either transfer into mitochondria for energy production or be secreted into circulation to influence metabolism in peripheral tissues. We therefore probed systemic changes in succinate. First, we profiled succinate levels in urine and feces over the first week after initiation of the DC_12_ diet. Urinary succinate approximately tripled by Day 7 of DC_12_ feeding (Figure 6F) in a manner that paralleled the rise of succinyl PTMs in liver and kidney. Interestingly, in feces, which are thought to reflect metabolism of the microbiome, succinate levels declined nearly 4-fold over the same 7-day period (Figure 6G). The mice were then maintained on the diet for 5 weeks, at which time serum, urine, and tissues were harvested during the dark cycle. Surprisingly, in chronic DC_12_-adapted animals, succinate levels were either significantly reduced or exhibited a nonsignificant trend in that direction (Figure 6H), arguing against the theory that succinate paracrine signaling contributes to the metabolic phenotype of DC_12_-fed animals. In the TCA cycle, Complex II (succinate dehydrogenase) converts succinate to fumarate. In liver, although the absolute amount of succinate trended downward with DC_12_ feeding, the succinate: fumarate ratio (substrate: product pair for Complex II) was significantly higher (Figure 6I), which could be caused by an influx of succinate from peroxisomal degradation of DC_12_. It therefore seems likely that, in DC_12_-adapted animals, DC_12_-derived succinate is transferred into mitochondria and oxidized.

### The ACOX1a isoform is required for peroxisomal generation of succinyl-CoA from DC_12_.

**Figure 7:**
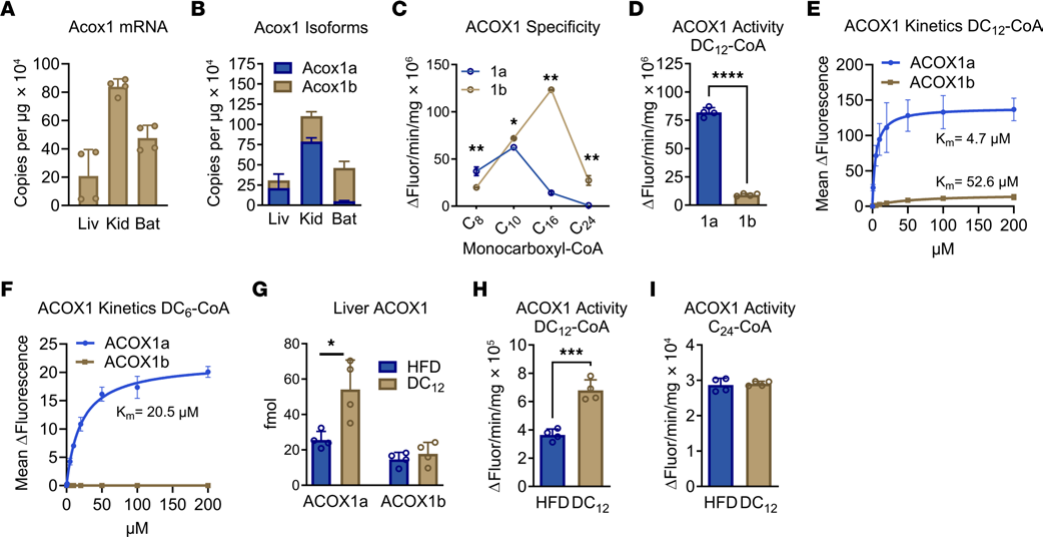
ACOX1a is required for peroxisomal generation of succinyl-CoA from DC_12_. (**A** and **B**) Digital droplet PCR was used for absolute quantification of total ACOX1 mRNA transcripts (**A**) and the 2 key isoforms ACOX1a and ACOX1b (**B**), expressed as copies per μg of RNA, in mouse liver (Liv), kidney (Kid), and BAT. (**C**–**F**) Characterization of recombinant human ACOX1a and ACOX1b enzyme activities with the indicated acyl-CoA substrates. Panels **C** and **D** were measured with 25 μM substrate. (**G**) Targeted proteomic assay employing parallel reaction monitoring was used to quantify the absolute amount of ACOX1a and ACOX1b in liver lysates of mice fed HFD versus DC_12_ diet. (**H** and **I**) ACOX1 enzyme activity (total activity, all isoforms) detected in liver lysates of mice on HFD versus an isocaloric DC_12_ diet. All graphs represent means and SDs. *P < 0.05, **P < 0.01, ***P < 0.001, ****P < 0.0001 as determined with 2-sided Student’s *t* test.

In the studies above, we observed that DC_12_-driven succinyl PTMs accumulate in tissues rich in peroxisomes, i.e., liver and kidney. A notable exception was BAT. BAT has been reported to express ACOX1 and to conduct robust peroxisomal FAO (45, 46), yet, in our DC_12_-fed mice, BAT did not demonstrate any increase in protein succinylation. We further investigated this paradox. Using droplet digital PCR (ddPCR) to quantify the absolute number of *Acox1* transcripts, we demonstrated that BAT expresses *Acox1* at levels higher than liver (Figure 7A). Immunoblotting confirmed ACOX1 protein in BAT (Supplemental Figure 7). However, there are 2 isoforms of the ACOX1 enzyme, dubbed ACOX1a and ACOX1b, which are equal in size and differ only in their use of an alternative exon 3. This unusual splicing of an alternative exon is conserved from zebrafish to humans and has been suggested to have functional ramifications (47–49). Using ddPCR assays specific to each isoform, we observed that *Acox1a* expression was high in liver and kidney, but not in BAT, which expressed predominantly *Acox1b* (Figure 7B). Next, we expressed and purified recombinant human ACOX1a and ACOX1b proteins and screened for enzyme activity against increasing chain lengths of monocarboxylic acyl-CoA substrates. ACOX1a exhibited maximal activity against the medium-chain substrate C_10_-CoA, while ACOX1b had maximal activity with the long-chain substrate C_16_-CoA. Perhaps most notably, only ACOX1b was measurably active against the very long-chain substrate C_24_-CoA (Figure 7C). Strikingly, ACOX1a showed nearly 10 × higher activity with DC_12_-CoA (Figure 7D). When the enzymes were assayed kinetically, the K_m_ of ACOX1a for DC_12_-CoA was observed to be under 5 μM compared with over 50 μM with ACOX1b (Figure 7E). Importantly, only ACOX1a has activity with DC_6_-CoA (Figure 7F), indicating that peroxisomal conversion of DC_6_-CoA to succinyl-CoA can only occur in tissues where ACOX1a is expressed. Finally, we observed that the ACOX1a isoform is specifically induced in liver by DC_12_ feeding. Stable isotope-labeled peptides representing the variable exon 3 region were synthesized and mass spectrometry was used to perform an absolute quantification of the 2 ACOX1 isoforms with or without DC_12_ feeding. The amount of the ACOX1a isoform doubled with DC_12_ feeding, while ACOX1b did not change (Figure 7G). In liver lysates, this corresponded to a doubling of ACOX1 enzyme activity with the ACOX1a substrate DC_12_-CoA and no change in enzyme activity with the ACOX1b substrate C_24_-CoA (Figure 7, H and I).

### DC_12_ does not compromise peroxisomal function.

**Figure 8:**
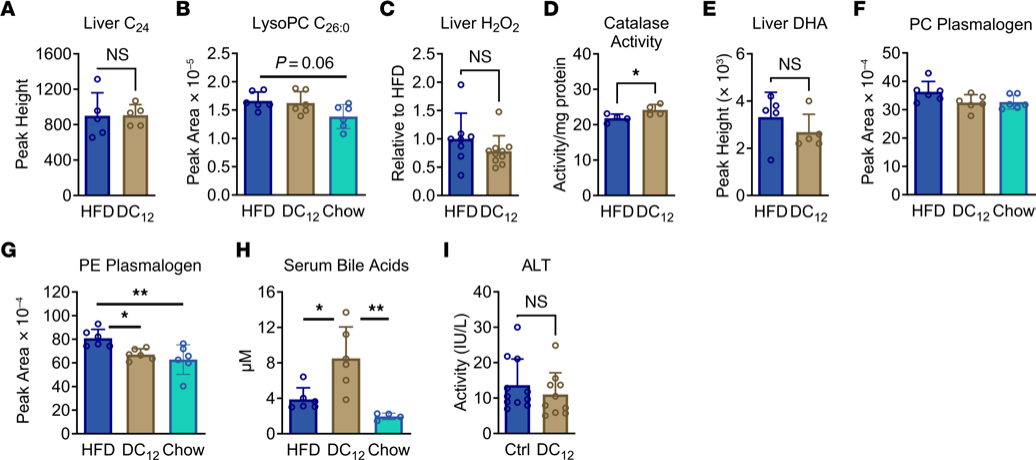
DC_12_ does not compromise peroxisomal function. (**A**–**D**) Peroxisomes contribute to degradation of very long–chain fatty acids (VLCFA) and H_2_O_2_ via catalase. Mass spectrometry was used to measure the VLCFA-related metabolites lignoceric acid (C_24_) in liver and lysophosphatidylcholine C_26:0_ in serum, in mice adapted chronically (5 wk) to HFD or an isocaloric DC_12_ diet. The amount of H_2_O_2_ was measured in snap-frozen liver, as was catalase enzyme activity. (**E**–**H**) Peroxisomes contribute to synthetic pathways for DHA, plasmalogens, and bile acids. Mass spectrometry was used to detect these lipid species in liver tissue (DHA) or serum (plasmalogens, bile acids). Panel **F** is the sum of 8 phosphatidylcholine plasmalogen species, panel **G** is the sum of 6 phosphoethanolamine plasmalogen species, and panel **H** is the sum of 11 primary C_24_ bile acids (conjugated and unconjugated). See Supplemental Tables 9 and 10 for serum plasmalogen and bile acid data, respectively. (**I**) Serum alanine aminotransferase (ALT) was measured as an indicator of liver injury. All graphs represent means and SDs. Panels **B** and **F**–**H** were analyzed with 1-way ANOVA and Tukey-corrected multiple comparisons while remaining panels were analyzed with 2-sided Student’s *t* test. *P < 0.05, **P < 0.01.

We have shown that DC_12_ dramatically increases the abundance of succinyl PTMs in peroxisomes of both liver and kidney. In mitochondria, where protein succinylation has been best studied, an increase in succinyl PTMs has been linked to decreased function of multiple enzymes (44, 50, 51). In contrast to this, one report on ACOX1 succinylation in peroxisomes suggested that succinylation may induce a gain of function rather than a loss of function (52). To our knowledge, the effect of lysine succinylation on other peroxisomal FAO enzymes, or on peroxisomal function in general, has not been studied. We therefore examined several indicators of peroxisomal activity ± DC_12_ feeding. Many genetic peroxisomal disorders are characterized by an accumulation of very long-chain fatty acids and associated very long-chain metabolites, such as lysophosphatidylcholine (LPC) C_26:0_ (53, 54). In DC_12_-fed mice, the amount of lignoceric acid (C_24_) and LPC C_26:0_ were unaltered (Figure 8, A and B). Another important peroxisomal function is removal of cellular H_2_O_2_ via catalase. The amount of H_2_O_2_ trended downward in DC_12_-fed liver while the enzymatic activity of catalase, one of the most highly succinylated proteins identified in our mass spectrometry survey, was significantly increased (Figure 8, C and D). Peroxisomes also have several biosynthetic functions, such as docosahexaenoic acid (DHA) synthesis, plasmalogen synthesis, and bile acid synthesis. Levels of DHA were unaltered by DC_12_ feeding, as was the total amount of serum phosphatidylcholine plasmalogens (Figure 8, E and F, and Supplemental Table 9). Serum phosphoethanolamine plasmalogens were significantly elevated in HFD-fed mice compared with either DC_12_ or chow-fed mice (Figure 8G and Supplemental Table 9). Finally, total serum C_24_ bile acids were altered by both HFD and DC_12_ diets. Total serum bile acids were significantly increased in DC_12_-fed mice compared with either the HFD or chow-fed groups (Figure 8H and Supplemental Table 10). Extreme increases (i.e., more than 100-fold) in serum C_24_ bile acids have been observed as a correlate of liver injury (55). The normal range of serum bile acids in mice is 1–20 μM (56), and thus the DC_12_-fed animals are well within in the normal range with a mean level of 8.5 μM. Indeed, we observed no sign of liver injury, as liver histology was normal (Supplemental Figure 8A) and serum levels of the injury marker alanine transaminase (ALT) were not altered by DC_12_ diet (Figure 8I). Chronically elevated bile acids can also induce damage to the heart (57). Again, we saw no indication of cardiac injury in DC_12_-fed animals, as indicated by normal heart histology, trichrome staining, and TUNEL staining (Supplemental Figure 8, B–D).

## Discussion

The studies presented here demonstrate the unique metabolic consequences of chronically consuming DC_12_. Our data are consistent with a model of action in which DC_12_ must be oxidized as it is being consumed since it cannot be stored. While the postprandial hormonal milieu promotes the storage of typical monocarboxylic dietary fat, DC_12_ remains “invisible” and is not seen as fat. In our indirect calorimetry studies, DC_12_-fed mice exhibited elevated respiration (VO_2_, VCO_2_) during the night while feeding on DC_12_, compared with either chow-fed or HFD-fed mice. This translated to a higher rate of energy expenditure during the night. Over time this increased rate of energy expenditure limited body fat stores, which, in turn, prevented declines in glucose sensitivity and muscle mitochondrial function. In essence, DC_12_-fed mice retained the body composition and glucose sensitivity of chow-fed controls, despite consuming 33% of calories as fat. In other words, it is not that DC_12_-fed mice had improved glucose tolerance, but rather, they failed to develop the signs of insulin resistance present in the HFD control group. Some limitations of our study must be noted with regards to glucose metabolism. First, we did not perform either hyperglycemic or hyperinsulinemic clamp studies to definitively assess glucose metabolism and insulin resistance. Second, we did not analyze for effects of DC_12_ on the microbiome, which could have contributed to glucose homeostasis (58). These limitations will be addressed in future studies.

One question that remains unanswered in our study is the fate of the excess energy expended during the dark cycle in DC_12_-fed animals. We tracked locomotion during our indirect calorimetry experiments and no change was seen in physical activity, demonstrating that activity is not the fate of the expended energy. The most likely explanation is that the energy is being converted to body heat. We attempted to address this by measuring core body temperature during the dark cycle, which revealed no change in the DC_12_-fed animals. However, our measures were made with a rectal thermometer, which may not have been a sensitive enough instrument and requires restraint of the animal, which could rapidly alter core temperature via a stress response. Proper interrogation of heat generation would require future experiments with implanted wireless temperature sensors. Heat production may be particularly robust in peroxisome-rich liver and kidney. Textbook dogma states that peroxisomal FAO does not produce energy — since peroxisomes lack an electron transport chain — but the energy released must be conserved, most likely as heat. The generation of heat by respiring peroxisomes has been both calculated (59) and experimentally demonstrated (60) and is an intriguing concept that will require future investigation.

**Figure 9:**
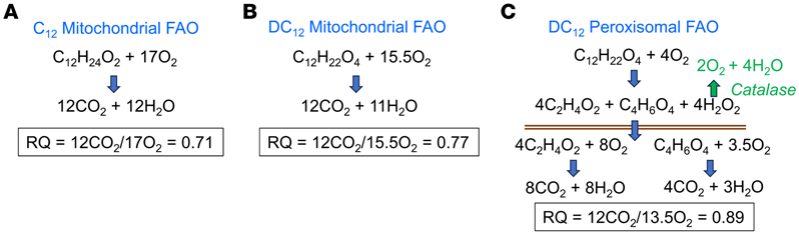
Respiratory quotients of 12-carbon fatty acids. (**A**) The respiratory quotient (RQ) of monocarboxylic C_12_ (lauric acid) oxidized to completion by mitochondria has an RQ of 0.71. (**B**) DC_12_, having 2 more oxygen molecules and 2 less hydrogens than C_12_, has an RQ of 0.77. (**C**) If DC_12_ is “predigested” by peroxisomes to acetate and succinate (above the brown lines), the oxygen requirement is reduced by 2 due to catalase reclaiming half of the oxygen used by ACOX1 in the peroxisome. This shifts the RQ up to 0.89.

While we believe that DC_12_ is immediately oxidized as it is being consumed, with the energy largely released as heat, our RER data calculated from indirect calorimetry were not consistent with this supposition. It is generally assumed that as FAO increases, RER decreases, due to the low respiratory quotient (RQ; the ratio of CO_2_ formed over O_2_ consumed) of fatty acids (approximately 0.70). However, our DC_12_-fed mice exhibited an RER of nearly 0.98 during the dark-cycle, which was similar to mice on low-fat chow and significantly higher than mice on HFD. This led us to examine the theoretical RQ of DC_12_ versus monocarboxylic fatty acids. Using the formula RQ = x/(x + y/4 - z/2), where x = carbon, y = hydrogen, and z = oxygen atoms, the predicted RQ of C_12_ (lauric acid) is 0.71 (Figure 9A). In comparison, the calculated RQ of DC_12_ oxidized by mitochondria is 0.77 (Figure 9B), because it contains 2 more oxygen atoms and 2 less hydrogens. However, in liver and kidney, where peroxisomes “predigest” DC_12_ down to succinate and acetate for subsequent oxidation by mitochondria, the calculation is more complex. Peroxisomal chain-shortening of DC_12_ by 4 rounds consumes 4 O_2_ at the step catalyzed by ACOX1, producing H_2_O_2_. However, due to the actions of catalase, 2 of the 4 O_2_ are reclaimed, which is enough to increase the RQ considerably, from 0.77 to 0.89 (Figure 9C). These 2 factors — the chemical composition of DC_12_ (more oxygen, less hydrogen) and the actions of peroxisomal catalase — likely combine to shift the RER upward in mice on DC_12_ diet compared with HFD.

**Figure 10:**
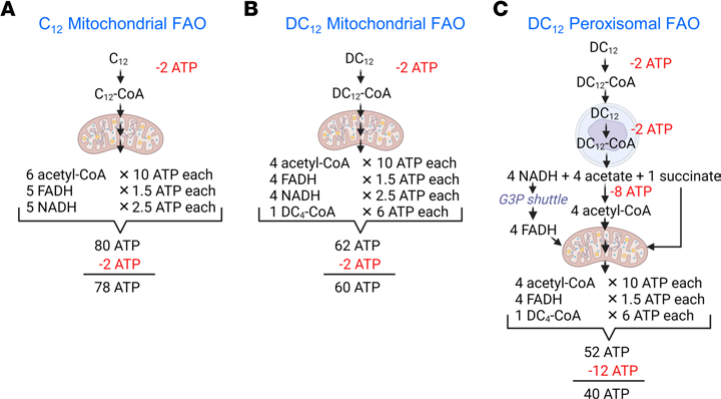
Metabolic efficiency of DC_12_ oxidation. (**A**) The theoretical yield of ATP from oxidizing monocarboxylic C_12_ through the mitochondrial FAO pathway and TCA cycle. Activation of fatty acids to CoA converts ATP to AMP, which is the energetic equivalent of 2 ATP (shown in red font). (**B**) Oxidizing DC_12_ through mitochondria yields a remnant succinate molecule, and less acetyl-CoA, FADH, and NADH. Therefore, the ATP yield is 23% lower than for C_12_ through the same pathway (60 versus 78). Finally, (**C**) oxidizing DC_12_ through peroxisomes, then passing the succinate, acetate, and NADH into mitochondria for complete oxidation requires a much greater cost of fatty acid activation, since each acetate must be activated to CoA at the cost of 2 ATP. The result is a nearly 50% reduction in net ATP compared with C_12_ in panel **A**. Image created with BioRender.com.

Another way to conceptualize the effect of DC_12_ on metabolic rate is to calculate the efficiency of DC_12_ oxidation versus “normal” fatty acids in terms of ATP production. Lauric acid (C_12_) oxidized by mitochondrial FAO would yield 80 ATP. However, the acyl-CoA synthetases that activate C_12_ to C_12_-CoA prior to catabolism hydrolyze ATP to AMP, which is the bioenergetic equivalent of 2 ATP. Thus, the net ATP yield for C_12_ oxidation is 78 ATP (Figure 10A). In comparison, oxidizing DC_12_ to completion in mitochondria (as we propose is done by muscle, heart, etc.) yields nearly 25% less energy because chain shortening would stop at succinyl-CoA, producing less acetyl-CoA and fewer reducing equivalents (Figure 10B). Finally, “predigesting” DC_12_ through peroxisomes would be even less bioenergetically efficient (Figure 10C). Activating DC_12_ to DC_12_-CoA in the cytosol would cost 2 ATP. Then, recent evidence indicates that it would need to be activated a second time after entry into peroxisomes (61, 62). After 4 rounds of chain shortening in the peroxisome, 4 acetate and 1 succinate would be released to the cytoplasm. Succinate can directly enter the TCA cycle, either locally or after transport to a distant tissue. Acetate, whether oxidized locally or released and oxidized elsewhere, ultimately needs to be reactivated to CoA at a cost of 2 ATP per acetate. Also, 4 NADH produced within the peroxisome would be shuttled across the peroxisomal membrane to produce 4 NADH in the cytoplasm. These are likely oxidized through the glycerol-3-phosphate (G3P) shuttle localized to the mitochondrial membrane, which converts them to FADH at an energetic cost of 1 ATP per NADH. Completing the math, DC_12_ may yield either 40 or 42 ATP depending on whether the original DC_12_ is activated to CoA twice or just once. In short, the metabolism of DC_12_ in the liver during feeding may produce only half as much energy as metabolizing a monocarboxylic fatty acid of the same length. This inefficiency may contribute to obesity prevention in DC_12_-fed mice.

We entered these studies with the hypothesis that DC_12_ would be chain-shortened to succinyl-CoA with subsequent conversion to succinate, which would then circulate and mimic the phenotype observed when mice are provided with exogenous succinate. However, we did not see any increase in succinate in serum, tissues, or urine from chronically fed animals, despite evidence supporting succinyl-CoA formation in liver, kidney, and, to a lesser extent, WAT. Rather, succinate tended to be decreased throughout the body. The only phenotype consistent with those reported for mice consuming oral succinate was “beiging” of WAT, evidenced by increased expression of the mitochondrial marker HSP60 and also UCP1. Protein succinylation was also increased in WAT, which would not occur if succinate was stimulating “beiging” from the outside of the adipocyte through the succinate receptor GPR91. It therefore seems that in the case of DC_12_ feeding, any succinate effect in WAT is produced locally within the adipocytes through DC_12_ β-oxidation.

A limitation of our studies that should be noted is that, while we demonstrate that tissues with a paucity of peroxisomes such as muscle and heart can metabolize DC_12_ through mitochondria in vitro, we were unable to determine the relative contribution of these organs to dietary DC_12_ disposal compared with liver and kidney. Muscle, heart, and brain all exhibited significant increases in steady-state DC_6_ levels in vivo but demonstrated no change in protein succinylation, which was a prevalent phenotype in liver and kidney. This could be because the mitochondrial FAO pathway is not capable of chain-shortening DC_6_ to DC_4_, or it could reflect a very low level of flux that is insufficient to increase the succinyl-CoA pool. Another possibility is that the lysine desuccinylase sirtuin-5 (SIRT5) efficiently counters any change in mitochondrial protein succinylation induced by DC_12_ in tissues like muscle and heart. Additionally, while our data measuring FAO flux in hepatocytes ± etomoxir indicated that a substantial fraction of DC_12_ was flowing through the mitochondrial pathway in liver, this may be an overestimation. Etomoxir is routinely used as an irreversible inhibitor of CPT1 but it may also inhibit related peroxisomal carnitine acyltransferase enzymes, such as carnitine octanoyltransferase (CROT) and carnitine acetyltransferase (CrAT), which could affect peroxisomal FAO (63). Further studies are needed to distinguish between the contribution of peroxisomes and mitochondria at the whole-body level using genetic methods to separate the individual pathways.

Unlike mitochondria, very little is known about the effects protein acylation on peroxisomal function. Lysine succinylation was previously reported on peroxisomal proteins, including the FAO enzymes ACOX1 and EHHADH, in mass spectrometry surveys of liver PTMs (51). Chen et al. (52) further showed that SIRT5 can localize to peroxisomes, where it desuccinylates and reduces the activity of ACOX1. Mechanistically, it was suggested that succinylation improves the activity of ACOX1 by stabilizing the protein dimer. Here, we observed a dramatic increase in succinylation of ACOX1, with some lysine residues showing as much as a 20,000-fold increase in succinylation. There are 4 lysine residues in exon 3 of ACOX1a and 0 lysine residues in exon 3 of ACOX1b, but none of the 4 ACOX1a-specific lysine residues were succinylated. Thus, all of the identified ACOX1 succinylation sites were common to both isoforms. Activity of ACOX1b (C_24_-CoA) was unchanged in liver from DC_12_-fed mice, and while activity of ACOX1a (DC_12_-CoA) increased, this is most likely explained by the increased abundance of ACOX1a. However, our studies did not explore whether peroxisomal Sirt5 was countering any potential positive effects of lysine succinylation on ACOX1. This will need to be addressed in future studies. Further, our mass spectrometry survey did not measure the stoichiometry of lysine succinylation within peroxisomes. Based on the small peak heights for succinylated peroxisomal peptides detected in HFD-fed mouse liver, we postulate that the stoichiometry of this PTM in peroxisomes under normal conditions is very low. This makes sense given that ω-oxidation, which produces the peroxisomal succinyl-CoA required to drive succinylation, is a minor pathway under normal physiological conditions. Thus, even a 1,000-fold increase in the abundance of a given succinylated peroxisomal peptide may still be low in terms of absolute stoichiometry.

In summary, our studies indicate that exogenous DC_12_ forces its own catabolism by virtue of not being a substrate for lipid storage. In peroxisome-rich tissues such as liver and kidney, DC_12_ catabolism can proceed unregulated, as there is no peroxisomal equivalent of mitochondrial CPT1 restricting the flow of carbon into the peroxisome. Further, the higher RQ for DC_12_ (0.89 in liver and kidney and 0.77 in other organs versus 0.70 for monocarboxylic fatty acids like palmitate) may be beneficial during ischemic conditions, including exercise. This is supported by reduced blood lactate in DC_12_-fed mice after treadmill running (Figure 2I). Reduced oxygen demand while catabolizing DCAs may also explain our previous observation that DCAs protect against ischemic kidney injury (64). Additional studies are underway to optimize the dosing and formulation for oral DC_12_. Here, we utilized the free DC_12_ fatty acid, which exhibits low solubility but could conceivably be mixed into solid foodstuffs. Alternatively, while DCAs cannot be esterified into triglycerides naturally in the body, they can be chemically synthesized as triglycerides (65, 66). This formulation could allow a broader application into foods, drinks, or nutritional supplements designed to deliver DCAs as an alternative form of energy that increases energy expenditure and prevents adipose expansion.

## Methods

See Supplemental Methods for full experimental details.

### Sex as a biological variable.

Our experimental design was to compare the effects of DC_12_ to that of an isocaloric HFD. The physiological consequences of HFD are known to be blunted in female mice (13). Therefore, we performed the great majority of the experiments in male mice only. One exception was the finding that DC_12_ feeding reduces adipose tissue mass, which was replicated in females and is shown in Supplemental Figure 1. It is unknown whether the other findings reported here are relevant for female mice.

### Statistics.

Statistics were calculated in GraphPad Prism version 10.1.0. All 2-group comparisons were done with 2-tailed Student’s *t* tests, and 3-group comparisons with 1-way ANOVA followed by the Tukey’s multiple comparison test. The cutoff for statistical significance was set at *P* < 0.05. For proteomics, *P* values were corrected for multiple testing using the Storey method. Protein changes with a q value under 0.05 and absolute Log_2_(fold-change) greater than 0.58 were considered statistically significant.

### Study approval.

All animal protocols were approved by the University of Pittsburgh Institutional Animal Care and Use Committee (IACUC), and all experiments were conducted in accordance with the guidelines and regulations set forth in the Animal Welfare Act (AWA) and PHS Policy on Humane Care and Use of Laboratory Animals.

### Data availability.

Complete MS data sets have been uploaded to the Center for Computational Mass Spectrometry, to the MassIVE repository at UCSD, and can be downloaded using the following FTP link: ftp://massive.ucsd.edu/v05/MSV000092073/ or via the MassIVE website: https://massive.ucsd.edu/ProteoSAFe/dataset.jsp?task=5cee1b4c1dbe40adac9ea25b0876660f (as prompted, connect by checking “Guest” [MassIVE ID number: MSV000092073]; [ProteomeXchange ID: PXD042602]). All other data are either included in the supplement or are available from the authors upon request

## Author contributions

ESG conceptualized the studies. The manuscript was written by ESG with contributions from YZ, JB, SSS, and BS. ESG, BBZ, YZ, SSB, KJS, AVS, KSR, ACR and ASB performed the animal experiments with DC_12,_ prepared primary cells, and assisted with data analysis. JB, JR, SS, and BS conducted mass spectrometry proteomics, succinylomics, and targeted quantification of ACOX1 isoforms, and analyzed mass spectrometry data. SFD performed GS-MS for urine metabolites. SJM and SLG performed LC-MS for serum, urine, and fecal metabolites. KEP assisted with histological analyses and TUNEL staining. YZ, BBZ, SSB, SSL, AVS, SFD, JB and BS edited the manuscript.

## Supplementary Material

Supplemental data

Unedited blot and gel images

Supplemental tables 1-13

Supporting data values

## Figures and Tables

**Table 1:**
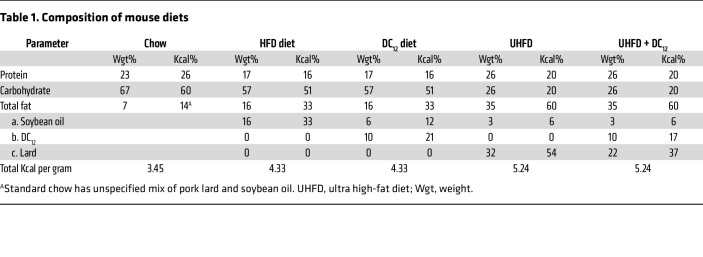
Composition of mouse diets

## Version 1 04/30/2024

In-Press Preview

## Version 2 06/17/2024

Electronic publication
