# Supplementary material for: Dietary dicarboxylic acids provide a nonstorable alternative fat source that protects mice against obesity
Source: J Clin Invest. 2024 Apr 30;134(12):e174186. doi: 10.1172/JCI174186 (PMC11178532; doi:10.1172/JCI174186)
Supplement: Supplemental data [file jci-134-174186-s051.pdf]

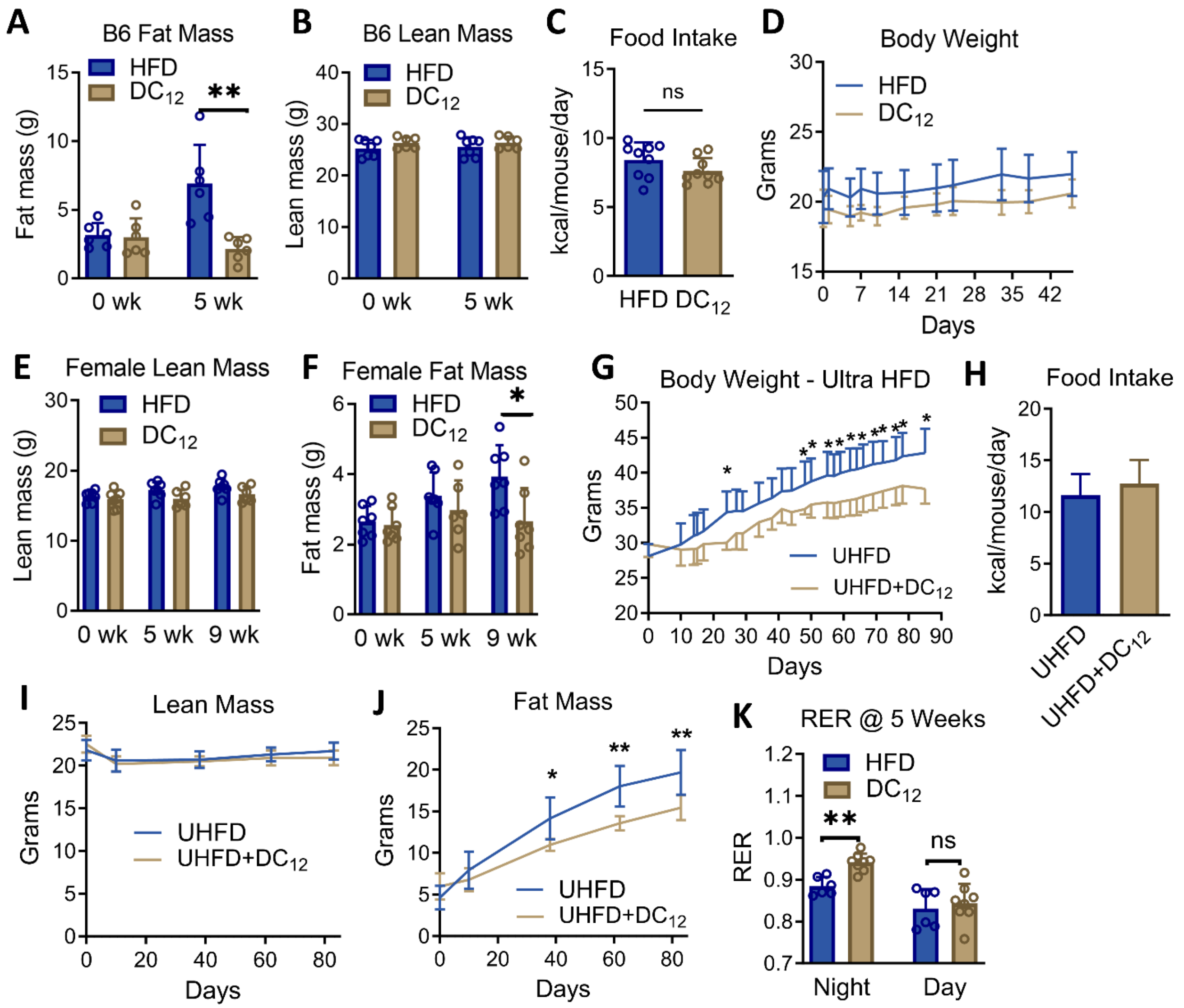

Supplemental Figure 1. DC_{12} protects against obesity. (A,B) EchoMRI of male C57BL/6 mice before and after 5 weeks of high-fat diet (HFD) versus an isocaloric DC_{12} diet (N=6). (C,D) Female C57BL/6 mice (N=6) were transitioned to the HFD and DC_{12} diets at age 8 weeks. Food pellets were weighed every 2-3 days to determine intake and body weights were recorded every 2-3 days. (E,F) EchoMRI was used to assess total fat mass and lean mass in B6 females after 5 weeks or 9 weeks. (G-J) Male 129S1 mice (N=8) were maintained on an ultra-high fat diet (UHFD, 60% calories from fat) or an isocaloric 60% fat diet containing 17% of calories from DC_{12} (UHFD+DC_{12}) for 80 days starting at age 8 weeks, with periodic EchoMRI measures. (K) Respiratory exchange ratios (RER) calculated from indirect calorimetry data on mice eating HFD or DC_{12} for 5 weeks. All graphs depict means and standard deviations. *P<0.05, **P<0.01, as determined with two-sided Student's t-test.

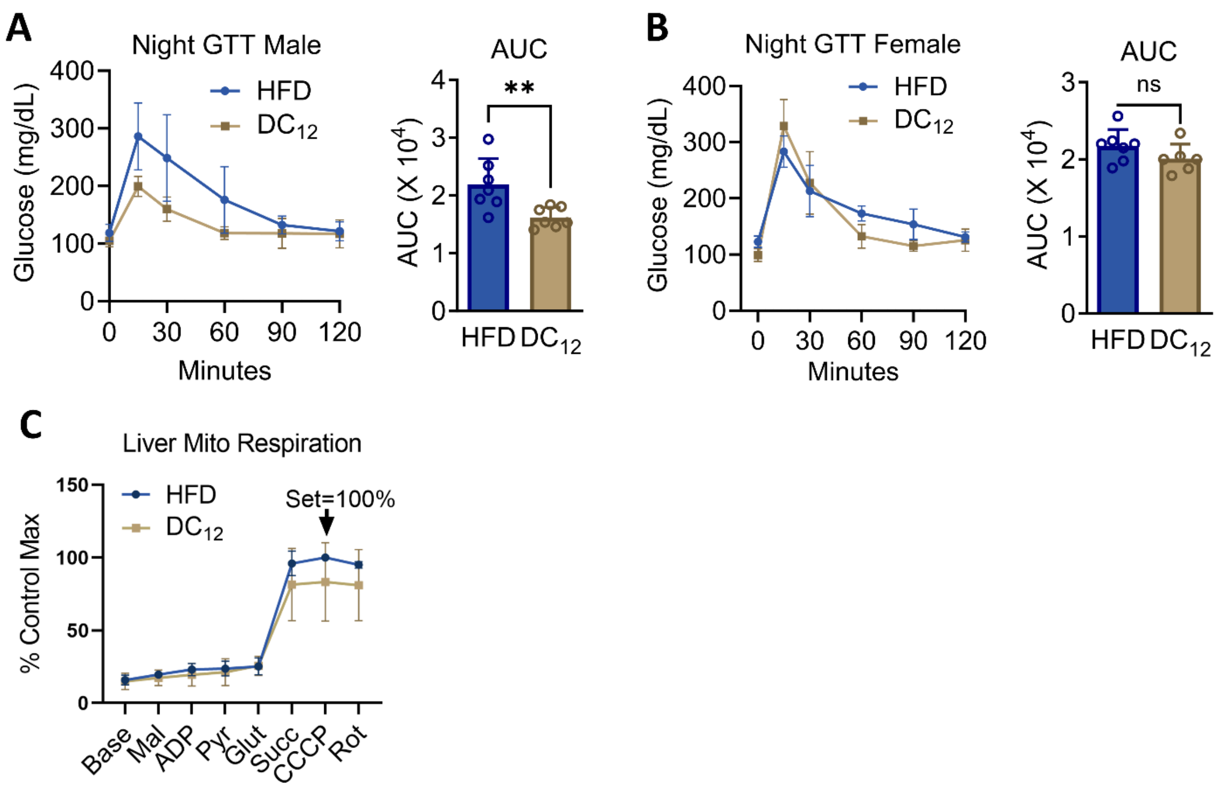

Supplemental Figure 2. DC_{12} diet maintains glucose sensitivity. (A) Fed male 129S1 mice maintained on either high-fat diet (HFD) or an isocaloric DC_{12} diet for 5 weeks were subjected to i.p. glucose tolerance testing (GTT) during the night. The area under the curve (AUC) is plotted in the bar graph. (B) Fed female C57BL/6 mice were subjected to i.p. GTT during the night and AUC calculated (C). Liver mitochondria isolated from mice on the special diets for 5 weeks were subjected to oroboros high-resolution respirometry (N=3). Maximum respiration in HFD mitochondria (in presence of uncoupler) was set=100% to facilitate combination of datasets across different days. All graphs depict means and standard deviations. *$P<0.05$, **$P<0.01$, as determined with two-sided Student's t-test.

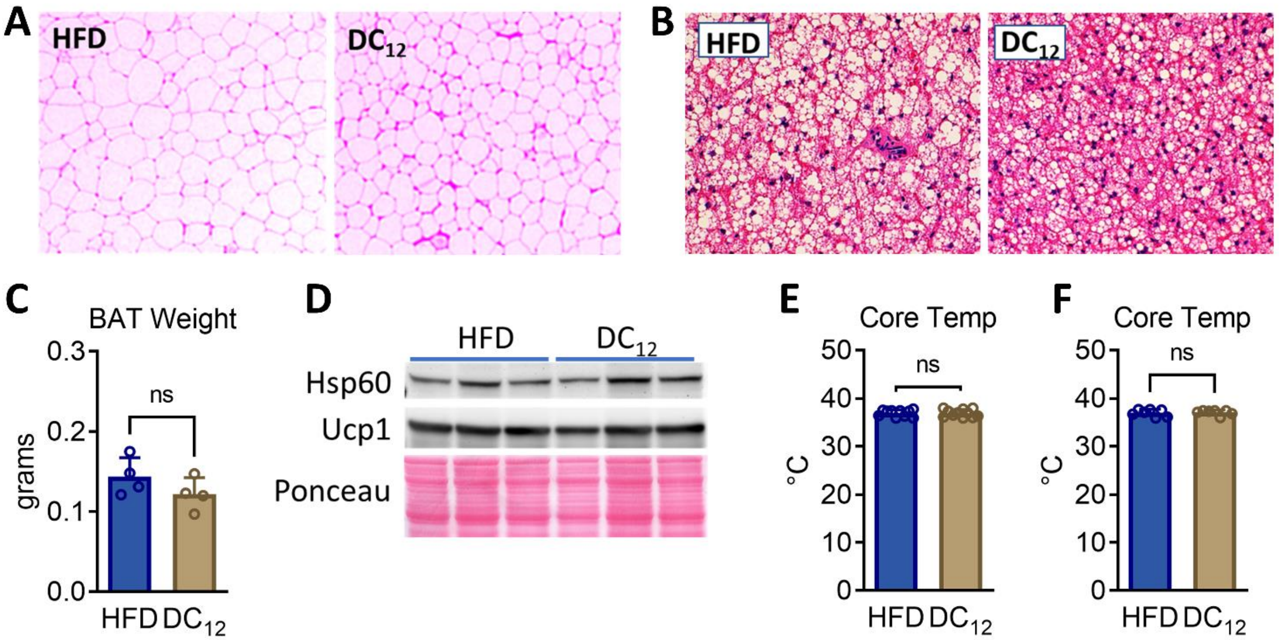

Supplemental Figure 3. Effect of DC_{12} on adipose tissues. (A,B) Hematoxylin & eosin stained inguinal white adipose tissue (panel A) and brown adipose tissue (BAT; panel B) after 5 weeks of high-fat diet (HFD) or an isocaloric DC_{12} diet. (C) BAT wet weight after 5 weeks of HFD or DC_{12} diet. (D), BAT immunoblotting for the mitochondrial marker heat-shock protein-60 (Hsp60) and uncoupling-protein-1 (Ucp1). (E,F) rectal temperatures in male (E) and female (F) 129S1 mice during the dark cycle, after 5 weeks of HFD or DC_{12} diet. All bar graphs represent means and standard deviations.

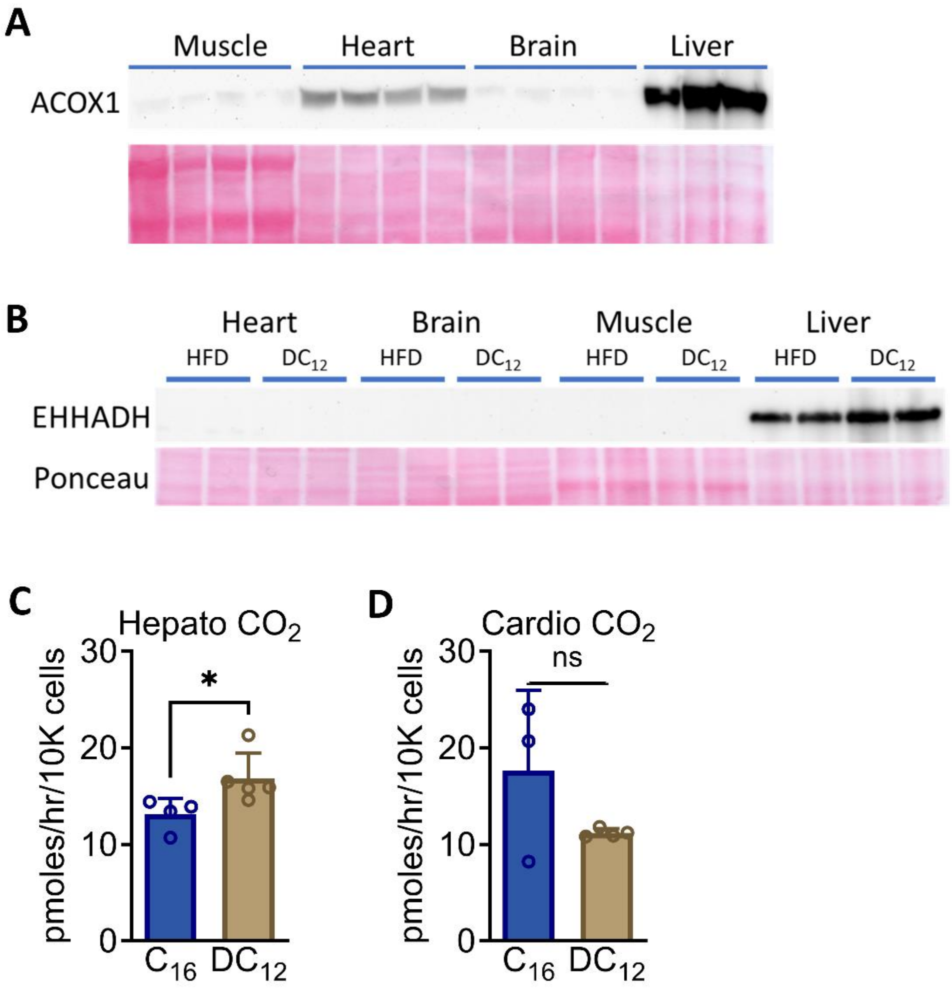

Supplemental Figure 4. **Expression of key peroxisomal FAO enzymes.** (A,B) 129S1 mice were fed high-fat diet (HFD) or an isocaloric DC_{12} diet for 5 weeks. Shown are immunoblots for acyl-CoA oxidase-1 (ACOX1) and enoyl-CoA hydratase/3-hydroxyacyl-CoA dehydrogenase (EHHADH) in total mouse tissue lysates, with ponceau staining as a loading control. Loadings were 45 µg per lane except for liver (15 µg). (C,D) Primary mouse hepatocytes and primary rat cardiomyocytes were incubated with ^{14}C-palmitate (C_{16}) or ^{14}C-DC_{12} for two hours and ^{14}C-CO_2 captured. All bar graphs represent means and standard deviations. *$P<0.05$.

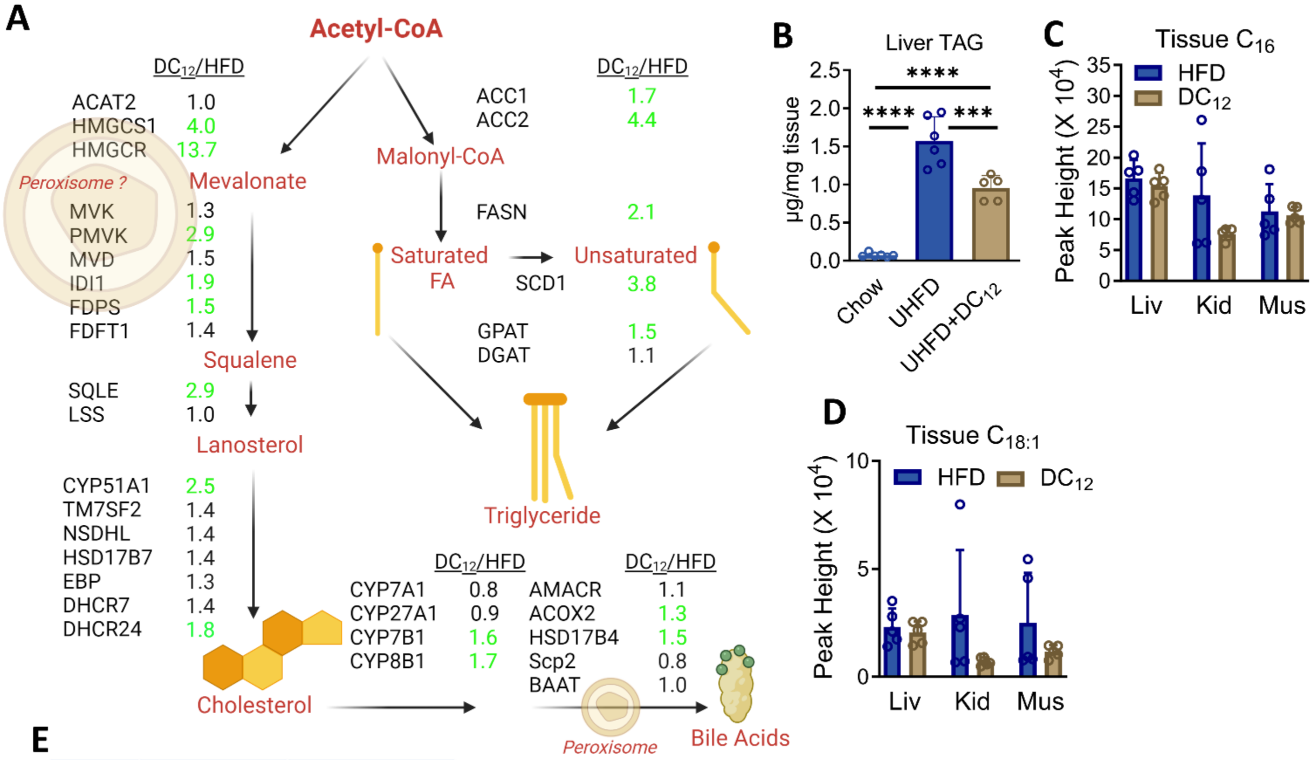

E

	*Cholesterol (mg/dL)	**Triglycerides (mg/dL)
HFD-1	104	<70
HFD-2	<100	161
HFD-3	124	92
HFD-4	152	133
DC12-1	<100	<70
DC12-2	<100	82
DC12-3	<100	94

**Cholesterol meter range: 100-400 mg/dL*

***Triglyceride meter range: 70-600 mg/dL*

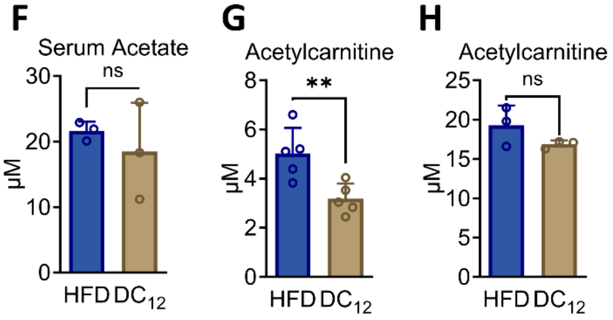

Supplemental Figure 5. Consumption of DC_{12} does not cause dyslipidemia or fatty liver. (A) Reactome pathway analysis of liver from DC_{12}-fed mice versus high-fat diet (HFD) mice indicated that lipid synthesis pathways were upregulated by DC_{12}. Shown in the cartoon are the fold-changes for individual proteins that were detected in these pathways. Green font indicates the change was statistically significant (q<0.01). Note that, while controversial, there is experimental evidence to indicate that the early steps of cholesterol synthesis from HMGCS1 to FDPS occur within peroxisomes. See Supplemental Tables 1 for more details and for full protein names. (B) Male 129S1 mice were fed an ultra-high fat diet (UHFD, 60% calories from fat), the same diet supplemented with DC_{12} (UHFD+DC_{12}), or standard laboratory chow for 9 weeks and liver was harvested for analysis of triglyceride (TAG) content. (C,D) Mass spectrometry was used to measure the most common saturated (C_{16}) and unsaturated ($C_{18:1}$) fatty acids in the indicated tissues after HFD or DC_{12} feeding for 5 weeks. (E) Handheld meters were used to estimate serum cholesterol and triglycerides during the dark cycle, after 5 weeks of HFD or DC_{12} diet, while (F) acetate was measured in serum using a colorimetric kit. (G,H) Mass spectrometry was used to measure acetylcarnitine in serum both at night (G) and during the day (H). All graphs depict means and standard deviations. **P<0.01, as determined with two-sided Student's t-test. ANOVA and Tukey's multiple comparison test were used to analyze panel B.****<0.0001. Panel A was made with BioRender.com.

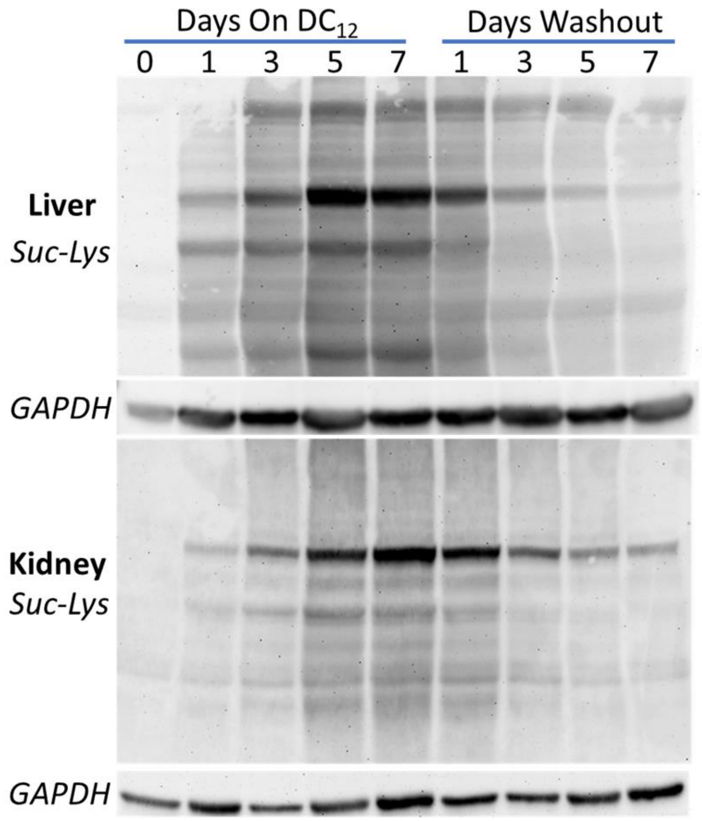

Supplemental Figure 6. Time course of protein succinylation during DC_{12} adaptation. Male 129S1 mice started a diet containing 10% w/w DC_{12} at Day 0. Liver (top) and kidney (bottom) were harvested on the indicated days, and 20 µg of total tissue lysates was immunoblotted with a pan anti-succinyllysine antibody (Suc-Lys). Similarly, after 7 days on the diet, the diet was replaced with standard chow and washout of the protein succinylation was followed for 7 days. GAPDH was immunoblotted as a loading control.

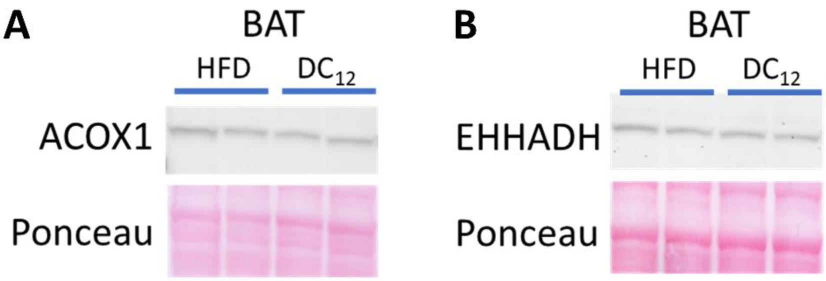

Supplemental Figure 7. Expression of key peroxisomal FAO enzymes in brown adipose tissue (BAT). Male 129S1 mice were maintained on either a high-fat diet (HFD) or an isocaloric DC_{12} diet for 5 weeks. BAT was harvested and immunoblotted for key enzymes that conduct the first 3 enzymatic steps of peroxisomal DC_{12} β-oxidation, which are acyl-CoA oxidase-1 (ACOX1) and enoyl-CoA hydratase/3-hydroxyacyl-CoA dehydrogenase (EHHADH). The diet did not influence expression of these proteins. Ponceau staining of the membranes serves as loading control.

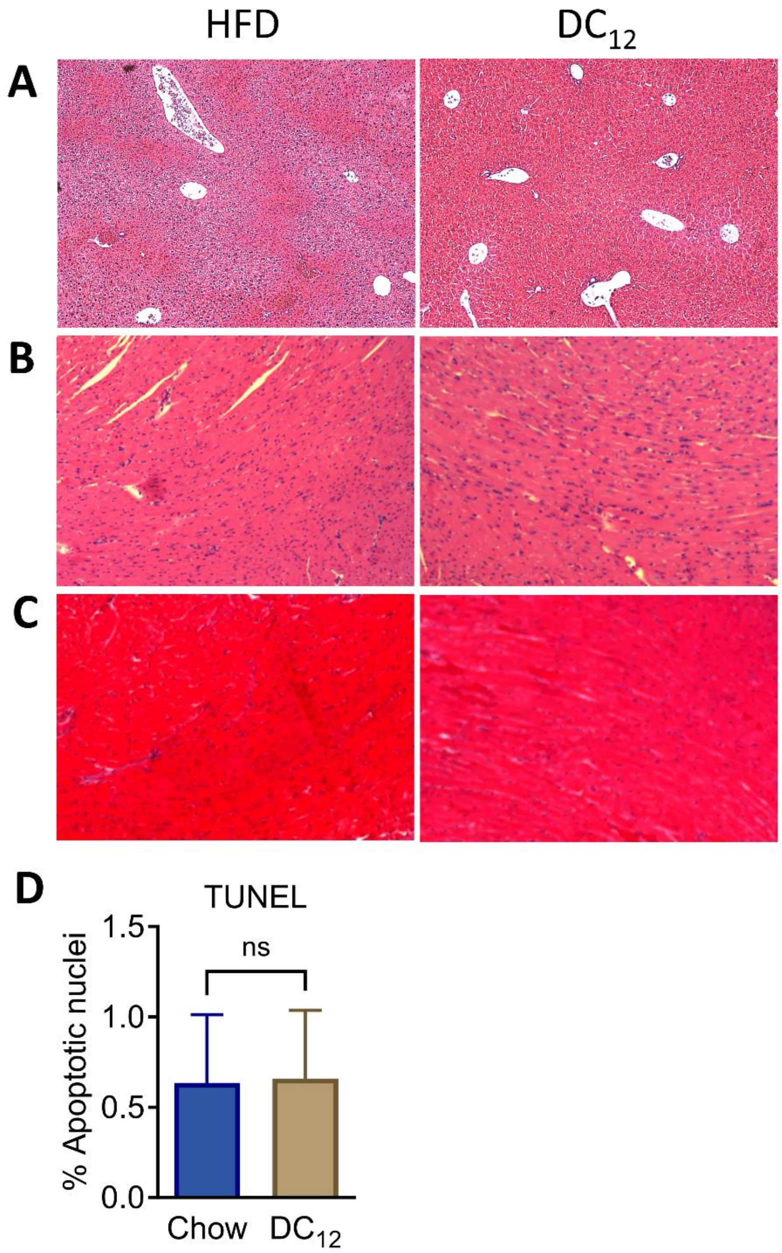

Supplemental Figure 8. Chronic DC_{12} feeding does not injure liver or heart. Mice were maintained on high-fat diet (HFD) or an isocaloric DC_{12} diet for 9 weeks, then tissue and serum were harvested. (A) Liver stained with hematoxylin and eosin. (B) Heart stained with hematoxylin and eosin. (C) Heart stained with Masson's trichrome as a marker of fibrosis. (D) Chow-fed and DC_{12}-fed mice were maintained on the diets for 9 weeks beginning at age 8 weeks. Apoptosis was assessed in cardiac tissue using TUNEL staining. Shown in the graph are means and standard deviations for the percentage of TUNEL-positive apoptotic nuclei.

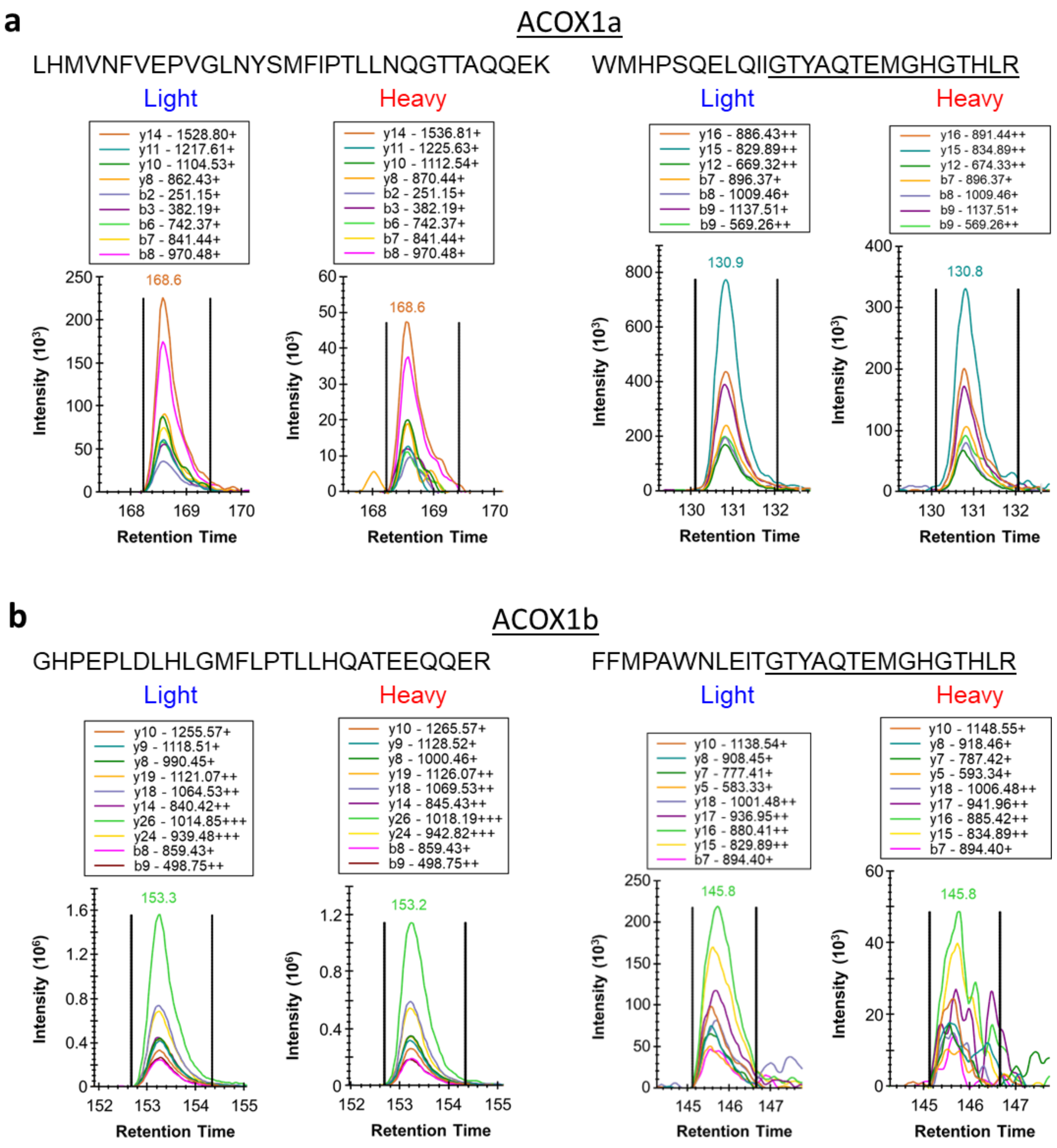

Supplemental Figure 9. **LC-PRM data of ACOX1 isoforms.** Extracted ion chromatograms of the endogenous (light) and reference synthetic (heavy-labelled) precursor ions of the mouse peroxisomal acyl-coenzyme A oxidase 1 (ACOX1, Q9R0H0) isoform specific peptide sequences in a DC_{12} replicate. LC-PRM data were processed with Skyline. a, ACOX1a-specific peptides LHMVNFVEPVGLNYSMFIPTLLNQGTTAQQEK (light: m/z 1207.28, z = 3+; heavy: m/z 1209.95, z = 3+) and WMHPSQELQIIGTYAQTEMGHGTHLR (light: m/z 756.12, z = 4+; heavy: m/z 758.62, z = 4+). b, ACOX1b-specific peptides GHPEPLDLHLGMFLPTLLHQATEEQQER (light: m/z 809.91, z = 4+; heavy: m/z 812.41, z = 4+) and FFMPAWNLEITGTYAQTEMGHGTHLR (light: m/z 752.86, z = 4+; heavy: m/z 755.36, z = 4+).

Supplemental Methods

Animals and DCA administration

All animal protocols were approved by the University of Pittsburgh Institutional Animal Care and Use Committee (IACUC), and all experiments were conducted in accordance with the guidelines and regulations set forth in the Animal Welfare Act (AWA) and PHS Policy on Humane Care and Use of Laboratory Animals. All mice were maintained on a 12 hr light/dark cycle in a pathogen-free barrier facility. 129S1 and C57BL/6J mice, age 8 weeks, were purchased from Jackson Laboratories (Bar Harbor, ME) and acclimated for 72 hr before introducing experimental diets. The DC_{12} diets and matching isocaloric high-fat diets were manufactured by Research Diets, Inc. Diet product numbers were: D21011101 (33% fat “HFD”); D20120406 (DC_{12}); D12492 (60% “ultra HFD”); and D22100403 (ultra HFD with DC_{12}). The DC_{12} diets were both 10% DC_{12} free acid by weight (Sigma-Aldrich Co., St. Louis, MO), as detailed in Table 1. Food intake was monitored by weighing the pelleted diet 3-4 times per week for 5 weeks. For the immunoblot experiments evaluating the accumulation and “washout” of protein succinylation, DC_{12} was mixed into powdered standard rodent diet and fed for the indicated time frames.

Whole-animal phenotyping

Body weight was monitored with a digital scale, with animals weighed early in the light cycle. Body composition was determined using the EchoMRI-100 system (Echo Medical Systems, Houston, TX), and indirect calorimetry was done in the Comprehensive Lab Animal Monitoring System (CLAMS, Columbus Instruments, Columbus, OH). For CLAMS, mice were acclimated to the special caging for 24-48 hr prior to a 48 hr monitoring period. Glucose tolerance testing was done either after a 5-hr fast (fasting GTT, Figure 2) during the light cycle or in non-fasted animals during the dark cycle (Nighttime GTT, Supplemental Figure 2). Five µl per gram body weight of 40% dextrose was injected i.p. and glucose monitored every 15 min. Tail-vein blood samples

were also collected for serum insulin measures. Core body temperature was measured during the dark cycle using a rodent rectal temperature probe (Physitemps Instruments, Clifton, NJ). Acute exercise challenges were done using the Exer3/6 rodent treadmill (Columbus Instruments, Columbus, OH). Naïve mice were acclimated to the treadmill with the belt off for five minutes, followed by 5 minutes of walking (8 m/min) and five minutes of slow running at 10 m/min. Then, speed was increased at a rate of 0.5 m/min every minute up to 14 m/min and held there for 20 minutes. Finally, mice were subjected to a second acceleration phase (0.5 m/min increase per minute) until exhaustion. Blood glucose, lactate, ketones, total cholesterol, and triglycerides were measured with hand-held multi-test meters from Keto-check, Inc (Napa, CA) and Germaine Laboratories (San Antonio, TX). Serum insulin was measured by ELISA (Crystal Chem, Elk Grove Village, IL). Liver triglycerides were assayed as described (1), by digestion in ethanolic KOH and assaying for free glycerol at 540 nm.

Gas chromatography-mass spectrometry (GC-MS) for urinary DCAs

For analysis of urine DCAs in chronic DC_{12}-adapted mice, urine was collected during the dark cycle in wire-bottomed metabolic cages (12 hr) and subjected GC-MS as described (1). Briefly, 2-phenylbutyrate was added as an internal standard, then organic acids were extracted by sequential ethyl ether and acetoacetate extractions and derivatized with trimethylsilane. Data were normalized to the creatinine content of the urine.

Tissue metabolites by GC-MS

Tissue succinate, adipate, fumarate, cholesterol, and fatty acids were measured using the primary metabolism panel (GC-TOF mass spectrometry) at the West Coast Metabolomics Center, UC-Davis.

Fecal and urinary DCAs by liquid chromatography-mass spectrometry (LC-MS)

Fecal and urinary DCAs on samples collected during 7 days of diet adaptation were measured by a targeted LC-MS assay at the University of Pittsburgh Health Sciences Mass Spectrometry Core Facility as detailed below. Urine and feces were collected during 24-hr intervals using wire-bottomed metabolic cages, every-other-day during the 7-day period. The weight of the fecal pellets and the volume of urine excreted over each 24 hr period was recorded and used for calculating the absolute amount of DCAs in each sample.

Fecal Sample Preparation for LC-MS

Fecal samples were homogenized with 50% aqueous acetonitrile at a ratio of 1:15 vol:wt. 5µg/mL Deuterated internal standards: (D2)-formate, (D4)-acetate, (D5)-butyrate, (D6)-propionate, (D2)-valerate and (D4)-hexanoate (CDN Isotopes, Quebec, Canada) were added. Calibration curves were similarly prepared from 33µg/mL down to 15ng/mL. Samples were homogenized using a FastPrep-24 system (MP-Bio), with Matrix D at 60hz for 30 seconds, before being cleared of protein by centrifugation at 16,000xg. 60µL cleared supernatants were collected and derivatized using 3-nitrophenylhydrazine (2). Each sample was mixed with 20 µL of 200 mM 3-nitrophenylhydrazine in 50% aqueous acetonitrile and 20 µL of 120 mM N-(3-dimethylaminopropyl)-N0-ethylcarbodiimide -6% pyridine solution in 50% aqueous acetonitrile. The mixture was reacted at 50°C for 40 minutes and the reaction was stopped with 0.45 mL of 50% acetonitrile.

Urine Sample Preparation for LC-MS

Urine samples were diluted 1:1 vol:vol with acetonitrile. 5µg/mL Deuterated internal standards: (D2)-formate, (D4)-acetate, (D5)-butyrate, (D6)-propionate, (D2)-valerate and (D4)-hexanoate (CDN Isotopes, Quebec, Canada) were added. Calibration curves were similarly prepared from 100µg/mL down to 15ng/mL. Samples were homogenized using a FastPrep-24 system (MP-Bio), with Matrix D at 60hz for 30 seconds, before being cleared of protein by centrifugation at

16,000xg. 60µL cleared supernatants were collected and derivatized using 3-nitrophenylhydrazine. Each sample was mixed with 20 µL of 200 mM 3-nitrophenylhydrazine in 50% aqueous acetonitrile and 20 µL of 120 mM N-(3-dimethylaminopropyl)-N0-ethylcarbodiimide -6% pyridine solution in 50% aqueous acetonitrile. The mixture was reacted at 50°C for 40 minutes and the reaction was stopped with 0.45 mL of 50% acetonitrile.

<u>LC-MS Analysis</u>

Derivatized samples were injected (50 µL) via a Thermo Vanquish UHPLC and separated over a reversed phase Phenomenex Kinetex 150mm x 2.1mm 1.7µM particle C18 column maintained at 55°C. For the 20 minute LC gradient, the mobile phase consisted of the following: solvent A (water / 0.1% FA) and solvent B (ACN / 0.1% FA). The gradient was the following: 0-2min 15% B, increase to 60%B over 10 minutes, continue increasing to 100%B over 1 minute, hold at 100%B for 3 minutes, reequillibrate at 15%B for 4 minutes. The Thermo IDX tribrid mass spectrometer was operated in both positive ion mode, scanning in ddMS2 mode (2 µscans) from 75 to 1000 m/z at 120,000 resolution with an AGC target of 2e5 for full scan, 2e4 for ms2 scans using HCD fragmentation at stepped 15,35,50 collision energies. Source ionization setting was 3.0kV spray voltage respectively for positive mode. Source gas parameters were 45 sheath gas, 12 auxiliary gas at 320°C, and 3 sweep gas. Calibration was performed prior to analysis using the PierceTM FlexMix Ion Calibration Solutions (Thermo Fisher Scientific). Integrated peak areas were then extracted manually using Quan Browser (Thermo Fisher Xcalibur ver. 2.7). DCA species to ITSD ratio were converted using the calibration curves and reported as ng/mg feces, or µM for urine concentration.

Serum phospholipids by LC-MS. The University of Pittsburgh Health Sciences Mass Spectrometry Core Facility measured serum plasmalogens by LC-MS as detailed below, following their previously published methods (3-6).

Sample preparation

Lipids were extracted by transferring 25µL serum to a clean glass tube containing 10µL LipidSplash and LYSO-PC deuterated lipid internal standards (Avanti Polar Lipids. Alabaster, AL), and subjected to a Folch extraction. Samples were rested on ice for 20 minutes before phase separation via centrifugation at 2500 x g for 20 minutes. 1.8mL of organic phase was dried to completed under nitrogen gas and resuspended in 100µL 1:1 acetonitrile:isopropanol. 3µL of sample was subjected to online LC-MS analysis.

LC-HRMS Method

Analyses were performed by untargeted LC-HRMS. Briefly, Samples were injected via a Thermo Vanquish UHPLC and separated over a reversed phase Thermo Accucore C-18 column (2.1×100mm, 5µm particle size) maintained at 55°C. For the 30 minute LC gradient, the mobile phase consisted of the following: solvent A (50:50 H2O:ACN 10mM ammonium acetate / 0.1% acetic acid) and solvent B (90:10 IPA:ACN 10mM ammonium acetate / 0.1% acetic acid). Initial loading condition is 30% B. The gradient was the following: Over 2 minutes, increase to 43%B, continue increasing to 55%B over 0.1 minutes, continue increasing to 65%B over 10 minutes, continue increasing to 85%B over 6 minutes, and finally increasing to 100% over 2 minutes. Hold at 100% for 5 minutes, followed by equilibration at 30%B for 5 minutes. The Thermo IDX tribrid mass spectrometer was operated in both positive and negative ESI mode. A data-dependent MS2 method scanning in Full MS mode from 200 to 1500 m/z at 120,000 resolution with an AGC target of 5e4 for triggering ms2 fragmentation using stepped HCD collision energies at 20,40,and 60% in the orbitrap at 15,000 resolution. Source ionization settings were 3.5 kV and 2.4kV spray voltage respectively for positive and negative mode. Source gas parameters were 35 sheath gas, 5 auxiliary gas at 300°C, and 1 sweep gas. Calibration was performed prior to analysis using the PierceTM FlexMix Ion Calibration Solutions (Thermo Fisher Scientific). Internal standard peak areas were then extracted manually using Quan Browser

(Thermo Fisher Xcalibur ver. 2.7), and used normalized analyte peak areas, then graphed using GraphPad PRISM (ver 9.0). Untargeted differential comparisons of lipid classes were performed using LipidSearch 4.2 (Thermo Fisher) to generate a ranked list of significant lipid compounds at the class and species-specific levels.

Serum bile acids by LC-MS. The University of Pittsburgh Health Sciences Mass Spectrometry Core Facility measured serum bile acids as previously described (7).

Sample Preparation

20µL of serum was transferred to a clean tube, 200µL of acetonitrile/3% HCl containing 160ng/mL Deuterated internal standards: D5- Tauroursodeoxycholic (TUDCA), D4-tauro-ß-muricholic (tßMCA), D5-taurodeoxycholic (TDCA), D4-glycochenodeoxycholic (GCGDA), D4-taurohyodeoxycholic- (THCA), D4-glycodeoxycholic (GDCA), D4-cholic (CA), D4-deoxycholic (DCA), D4-w-muricholic (wMCA), D4-glycolithocholic (GLCA), D4-glycocholic (GCA), D4-ursodeoxycholic (UDCA), D4- glycoursodeoxycholic (GUDCA), D4-α-muricholic (αMCA), D4-lithocholic (LCA), D4-chenodeoxycholic (CDCA), D4-taurochenodeoxycholic (TCDCA), D4-hyocholic (HCA), D5-taurolithocholic (TLCA), D5-ß-muricholic (ßMCA), D4-taurocholic (TCA), D4-tauro-αmuricholic (tαMCA), (Cayman Chemical, MI, USA) was added. Calibration curves of THCA, TßMCA, GHCA, 3-oxoCA, TUDCA, GUDCA, 7-keto-LCA, 7,12-diketo-LCA, ßMCA, TCA, GCA, UDCA, TCDCA, CA, GCDCA, TDCA, GDCA, TLCA, CDCA, GLCA, DCA, LCA were prepared alongside samples from 1µg/mL down to 50ng/mL. Samples were vortexed and sonicated in a water bath for 5 minutes, before being cleared of protein/precipitate by centrifugation at 16,000xg. 200µL cleared supernatant was dried to completion under N2 gas before resuspention in 40µL 1:1 water:MeOH and transferred to MS vials.

LC-MS Analysis

Samples were injected (5 µL) via a Thermo Vanquish UHPLC and separated over a reversed phase Phenomenex Luna 100mm x 2.1mm 3.0µM particle C18 column maintained at 55°C. For the 20 minute LC gradient, the mobile phase consisted of the following: solvent A (water / 5mM ammonium acetate) and solvent B (MeOH). The gradient was the following: 0-0.1min 52% B, increase to 85%B over 12.4 minutes, continue increasing to 100%B over 0.1 minute, hold at 100%B for 4 minutes, reequillibrate at 152%B for 4 minutes. The Thermo Exploris 240 mass spectrometer was operated in negative ion mode, scanning in ddMS2 mode (2 µscans) from 100 to 800 m/z at 120,000 resolution with an AGC target of 2e5 for full scan, 2e4 for ms2 scans using HCD fragmentation at stepped 25,35,50 collision energies. Source ionization setting was 3.5kV spray voltage. Source gas parameters were 50 sheath gas, 10 auxiliary gas at 325°C, and 1 sweep gas. Calibration was performed prior to analysis using the PierceTM FlexMix Ion Calibration Solutions (Thermo Fisher Scientific). Integrated peak areas were then extracted manually using Quan Browser (Thermo Fisher Xcalibur ver. 2.7). BA reported as area ratio of BA to the internal standard before conversion to concentration using calibration curves.

Immunoblotting

Tissue lysates were electrophoresed on Criterion SDS polyacrylamide gels (BioRad, Hercules, CA) and transferred to nitrocellulose membranes. Antibodies used were: anti-succinyllysine (PTM Biolabs PTM-401 and PTM-419); anti-acetyllysine (Cell Signaling #9681 and #9441); anti-acyl-CoA oxidase-1 (ACOX1; Abcam #ab59964); anti enoyl-CoA hydratase/3-hydroxyacyl-CoA dehydrogenase (EHHADH; Abcam # ab136059); anti-heat shock protein-60 (HSP60; Cell Signaling #12165); and anti-uncoupling protein-1 (UCP1; Abcam #ab234430). After incubation with HRP-conjugated secondary antibodies blots were visualized with chemiluminescence, and in some cases, scanned and subjected to densitometric analysis using ImageJ software.

Oroboros respirometry

Freshly isolated liver and muscle mitochondria were evaluated as previously described (8). Approximately 25 µg of intact mitochondria were added to 2 ml of Mir05 respiration medium. After the baseline became stable, 10 µM cytochrome c was added to assess mitochondrial integrity. Then, malate (2 mM), ADP (1.25 mM), pyruvate (5 mM), and glutamate (10 mM), and succinate (10 mM) were added to stimulate State 3 respiration.

Proteomics

Pieces of frozen liver, kidney, heart, muscle, and brain from the same HFD- (n=3) and DC_{12}-fed (n=3) mice were homogenized in a bead beater in a solution containing 5% SDS and 8 M urea. Samples were digested with trypsin in S-Trap mini columns (Protifi, Farmingdale, NY) followed by peptide desalting with Oasis 10-mg Sorbent Cartridges (Waters, Milford, MA). LC-MS/MS analyses were performed on a Dionex UltiMate 3000 system online coupled to an Orbitrap Eclipse Tribrid mass spectrometer (Thermo Fisher Scientific, San Jose, CA). All samples were acquired in data-independent acquisition (DIA) mode (9-11). Briefly, full MS spectra were collected at 120,000 resolution (AGC target: 3e6 ions, maximum injection time: 60 ms, 350-1,650 m/z), and MS2 spectra at 30,000 resolution (AGC target: 3e6 ions, maximum injection time: Auto, NCE: 27, fixed first mass 200 m/z). The DIA precursor ion isolation scheme consisted of 26 variable windows covering the 350-1,650 m/z mass range with an overlap of 1 m/z(11) (Supplemental Table 11).

DIA data processing and statistical analysis were performed in Spectronaut (version 17.3.230224.55965; Biognosys, Schlieren, Switzerland) using directDIA. Data was searched against all *Mus musculus* protein entries extracted from UniProtKB-TrEMBL (58,430 entries, 01/31/2018). Trypsin/P was set as digestion enzyme and two missed cleavages were allowed. Cysteine carbamidomethylation was set as fixed modification, and methionine oxidation and protein N-terminus acetylation as variable modifications. Data extraction parameters were set as dynamic and non-linear iRT calibration with precision iRT was selected. Identification was

performed using 1% precursor and protein q-value. Quantification was based on the extracted ion chromatograms (XICs) of 3 – 6 MS2 fragment ions, and local normalization was applied. Differential expression analysis was performed using an unpaired t-test, and p-values were corrected for multiple testing, specifically applying group wise testing corrections using the Storey method(12). Protein groups are required with at least two unique peptides, and protein changes with a q-value < 0.05 and absolute Log_2(fold-change) > 0.58 are required to qualify as 'significantly altered'. All quantification results are provided in Supplemental Tables 1-5.

Succinylomics

Pieces of frozen liver (~100 mg) or kidney (~50 mg) were homogenized, denatured, alkylated, trypsinized, and desalted as described (13, 14). 100 µg of each peptide elution was set aside for analysis of protein-level changes, and the remaining material used for enrichment of succinylated peptides with the PTMScan Succinyl-Lysine Motif Kit (Cell Signaling Technologies). LC-MS/MS analyses were performed on a Dionex UltiMate 3000 system online coupled to an Orbitrap Eclipse Tribrid mass spectrometer in DIA mode, as described above. DIA data was processed in Spectronaut (versions 14.10.201222.47784 and 15.1.210713.50606) using directDIA for both the protein level as well as PTM enriched samples, as described above. Data was searched against all *Mus musculus* protein entries extracted from UniProtKB-TrEMBL (58,430 entries, 01/31/2018, for the liver samples and 86,521 entries, 08/24/2021, for the kidney samples). For the PTM-enriched samples, lysine succinylation was additionally set as variable modification. PTM localization was selected with a probability cutoff of 0.75. Quantification was based on the XICs of 3 – 6 MS2 fragment ions, specifically b- and y-ions, without normalization, as well as data filtering using q-value sparse. Grouping and quantitation of PTM peptides were accomplished using the following criteria: minor grouping by modified sequence and minor group quantity by mean precursor quantity. Differential expression analysis was performed using a paired t-test, and p-values were corrected for multiple testing, specifically applying group wise

testing corrections using the Storey method (12). For the PTM analysis, each succinylated peptide was quantified individually, with significance established as q-value < 0.05 and absolute Log_2(fold-change) > 0.58. All PTM site identification and quantification results are provided in Supplemental Tables 7 and 8 (note: succinylation site changes are reported per each site with and without normalization to protein level changes). Subcellular localization was mapped using GO Cellular Component identifiers and verified manually by cross-referencing to MitoCarta 2.0 and PeroxisomeDB (15, 16).

ACOX1 isoform quantification using targeted assays

The ACOX1a and 1b isoforms arise through differential usage of two alternative versions of exon 3. The absolute mRNA copies of each isoform were quantified as follows. Freshly isolated mouse tissues were preserved in RNAlater Stabilization Solution (Invitrogen, AM7020) and total RNA was extracted using RNeasy Mini Kit (QIAGEN, 74104). cDNA synthesis was performed with equal amounts of total RNA using SuperScript III First-Strand Synthesis System (Invitrogen, 18080051). Droplet Digital PCR (ddPCR) assays were performed using the QX200 Droplet Digital PCR System (Bio-Rad, 1864001). Predesigned primers/probe mix for ACOX1a (Assay ID: Mm.PT.58.50503784), ACOX1b (Assay ID: Mm.PT.58.10805842) or total ACOX1 (Assay ID: Mm.PT.58.5874703) were purchased from Integrated DNA Technologies (see Supplemental Table 12 for sequences).

At the protein level, the two isoforms were quantified in liver (N=3) using targeted proteomics. Two differentiating peptides were chosen per ACOX1 isoform (UniProt IDs: Q9R0H0-1 and Q9R0H0-2): i) LHMVNFVEPVGLNYSMFIPTLLNQGTTAQQEK and WMHPSQELQIIGTYAQTEMGHGTHLR for ACOX1a, and ii) GHPEPLDLHLGMFLPTLLHQATEEQQER and FFMPAWNLEITGTYAQTEMGHGTHLR for ACOX1b. Selected peptides were synthetized with >98% purity and stable isotope-labelled C-terminal lysine ($^{13}C_6$, $^{15}N_2$) or arginine ($^{13}C_6$, $^{15}N_4$) residue (Biosynth). Liver lysates were used for

targeted parallel reaction monitoring assays (LC-PRM). A concentration-balanced mixture of the four accurately quantified stable isotope-labelled peptides (Biosynth) was spiked into all samples prior to LC-PRM analyses. LC-PRM analyses were performed on the same LC-MS system as for the DIA analyses (Orbitrap Eclipse). An unscheduled PRM method consisting of two full MS scans and 30 targeted MS2 scans was developed. The full MS scans were collected from 200-1,650 m/z at a resolution of 120,000 at 200 m/z (AGC target: 12e5, maximum IT: Auto). Targeted MS2 scans were collected at a resolution of 60,000 at 200 m/z (AGC target: 3e6, maximum IT: Auto, fixed first mass: 100 m/z). The 30 precursors were isolated within a 1.2-m/z window and fragmented with an NCE of 27 (Supplemental Table 13). PRM data were processed with Skyline (version 21.2.1.455)(17). Briefly, product ions (singly- to quadruply-charged y- and b-type ions) from ‘ion 2’ to ‘last ion – 1’ per precursor ion were extracted. All matching scans were used. Chromatographic peaks were investigated to manually adjust peak integration boundaries and remove interfered transitions. One to two precursor ions were kept per peptide, and 7-11 transitions per precursor ion. Signal at the precursor level was obtained by summing the corresponding transition peak areas, and at the peptide level by summing the corresponding precursor peak areas (Supplemental Fig 9).

ACOX1 enzyme activity assays

Acyl-CoA oxidase-1 (ACOX1) activity was assayed with 25 µM of acyl-CoA substrate and following H_2O_2 production with Amplex Red as described (18, 19). 20 µg of protein from a peroxisome-enriched liver fraction were added to 50 mM phosphate buffer pH 7.0 containing HRP (1 U/ml) and Amplex UltraRed (50 µM) at 30°C. Reactions were started by addition of acyl-CoA substrate and fluorescence was monitored for 30 minutes.

Primary cell isolation

For hepatocyte isolation, a 27-gauge butterfly needle attached to a 30 mL syringe was inserted into the Inferior Vena Cava (IVC) to begin liver perfusion with buffer. The Portal Vein (PV) was snipped to establish an open system. 80 mL of KREBS-Ringer Bicarbonate Buffer (Sigma-Aldrich Co., St. Louis, MO) was perfused through the system over the course of 10 mins. Then, 80 – 100 mL of KREBS-Ringer Bicarbonate Buffer containing 40 mg of collagenase type II (Sigma, St. Louis, MO) was perfused through the system for an additional 15 – 20 mins. Hepatocytes from the perfused liver were isolated in ice-cold DMEM (Sigma, St. Louis, MO) and passed through a 70 µm mesh filter. Cells were washed with 10 mL ice-cold DMEM (Sigma) before viable hepatocytes were separated using a 40% Percoll (Sigma-Aldrich, St. Louis, MO) solution gradient. Cells were washed in 10 mL of ice-cold DMEM (Sigma) and used immediately.

Adult cardiomyocytes were isolated from 10–12-week-old male Sprague Dawley rats on normal chow diets, following a modified Langendorff-free isolation protocol (20). The rats were anesthetized, and their chest walls were opened to expose the heart. The LV was injected with heparin to prevent coronary thrombosis, followed by clamping of the ascending aorta to facilitate retrograde perfusion. The hearts were excised with the clamped aortas into ice cold calcium free buffer. Digestion was performed at 37°C in a warm 10 cm^2 dish by successive injections of calcium free buffer containing collagenase type II (300 activity units/ml) via the LV apex. Following digestion, atrial tissue was separated away, and the ventricles were gently cut into 1 mm tissue pieces using forceps and triturated to complete enzymatic dissociation and release cardiomyocytes. Digested cell slurries were passed through individual 250 µm mesh filters and isolated cardiomyocytes were allowed to settle by gravity in conical tubes. Calcium was reintroduced to physiological levels (0.9mM) over 4 sequential rounds of intermediate concentrations accompanied by gravity settling. For Seahorse extracellular flux analysis, 15,000 cells were seeded per well onto laminin coated plates and allowed to equilibrate for thirty minutes prior to media change and analysis.

Fatty acid oxidation flux assays

1-^{14}C-labeled palmitate (C_{16}) and 1-^{14}C-dodecanedioc acid (DC_{12}) were sourced from PerkinElmer (Waltham, MA) and Moravek, Inc (Brea, CA), respectively. Just prior to assay, the fatty acids were dried to completion under nitrogen and then solubilized in 10 mg/ml α-cyclodextrin via incubation at 37°C for 30 min. Whole-cell assays with primary cells used ~100K cells in suspension, with a final fatty acid concentration of 50 µM and a 1 hr reaction time, ± 100 µM of etomoxir to inhibit the mitochondrial pathway. Whole-cell assays with differentiated myotubules were done in a six-well plate. At the end of the reaction, cells were scraped from the wells, lysed, and extracted for FAO products. FAO reactions with tissue lysates consisted of ~100 µg of lysate in a total reaction volume of 200 µl containing 100 mM sucrose, 10 mM Tris-HCl, 5 mM KH_2PO_4, 0.2 mM EDTA, 80 mM KCl, 1 mM $MgCl_2$, 0.1 mM malate, 0.05 mM coenzyme A, 2 mM ATP, 1 mM DTT, and 50 µM of fatty acid (0.5 µCi/ml). After 1 hr incubation at 37°C, FAO reactions were stopped by adding perchloric acid to 0.5 M final concentration. Following centrifugation to remove precipitated material, the water-soluble ^{14}C-labeled FAO products were isolated by methanol-chloroform extraction and quantified on a scintillation counter. The results were normalized to protein concentration.

References

1. Goetzman ES, Bharathi SS, Zhang Y, Zhao XJ, Dobrowolski SF, Peasley K, et al. Impaired mitochondrial medium-chain fatty acid oxidation drives periportal macrovesicular steatosis in sirtuin-5 knockout mice. *Scientific reports.* 2020;10(1):18367.
2. Han J, Lin K, Sequeira C, and Borchers CH. An isotope-labeled chemical derivatization method for the quantitation of short-chain fatty acids in human feces by liquid chromatography-tandem mass spectrometry. *Anal Chim Acta.* 2015;854:86-94.
3. Suber TL, Wendell SG, Mullett SJ, Zuchelkowski B, Bain W, Kitsios GD, et al. Serum metabolomic signatures of fatty acid oxidation defects differentiate host-response subphenotypes of acute respiratory distress syndrome. *Respir Res.* 2023;24(1):136.
4. Marron MM, Moore SC, Wendell SG, Boudreau RM, Miljkovic I, Sekikawa A, et al. Using lipid profiling to better characterize metabolic differences in apolipoprotein E (APOE) genotype among community-dwelling older Black men. *Geroscience.* 2022;44(2):1083-94.

5. Houston R, Sekine Y, Larsen MB, Murakami K, Mullett SJ, Wendell SG, et al. Discovery of bactericides as an acute mitochondrial membrane damage inducer. *Molecular biology of the cell.* 2021;32(21):ar32.
6. Thapa D, Xie B, Zhang M, Stoner MW, Manning JR, Huckestein BR, et al. Adropin treatment restores cardiac glucose oxidation in pre-diabetic obese mice. *J Mol Cell Cardiol.* 2019;129:174-8.
7. Manni ML, Heinrich VA, Buchan GJ, O'Brien JP, Uvalle C, Cechova V, et al. Nitroalkene fatty acids modulate bile acid metabolism and lung function in obese asthma. *Scientific reports.* 2021;11(1):17788.
8. Pereyra AS, Harris KL, Soepriatna AH, Waterbury QA, Bharathi SS, Zhang Y, et al. Octanoate is differentially metabolized in liver and muscle and fails to rescue cardiomyopathy in CPT2 deficiency. *Journal of lipid research.* 2021;62:100069.
9. Gillet LC, Navarro P, Tate S, Rost H, Selevsek N, Reiter L, et al. Targeted data extraction of the MS/MS spectra generated by data-independent acquisition: a new concept for consistent and accurate proteome analysis. *Molecular & cellular proteomics : MCP.* 2012;11(6):O111 016717.
10. Collins BC, Hunter CL, Liu Y, Schilling B, Rosenberger G, Bader SL, et al. Multi-laboratory assessment of reproducibility, qualitative and quantitative performance of SWATH-mass spectrometry. *Nature communications.* 2017;8(1):291.
11. Bruderer R, Bernhardt OM, Gandhi T, Xuan Y, Sondermann J, Schmidt M, et al. Optimization of Experimental Parameters in Data-Independent Mass Spectrometry Significantly Increases Depth and Reproducibility of Results. *Molecular & cellular proteomics : MCP.* 2017;16(12):2296-309.
12. Burger T. Gentle Introduction to the Statistical Foundations of False Discovery Rate in Quantitative Proteomics. *Journal of proteome research.* 2018;17(1):12-22.
13. Chiba T, Peasley KD, Cargill KR, Maringer KV, Bharathi SS, Mukherjee E, et al. Sirtuin 5 Regulates Proximal Tubule Fatty Acid Oxidation to Protect against AKI. *J Am Soc Nephrol.* 2019;30(12):2384-98.
14. Wei L, Meyer JG, and Schilling B. Quantification of Site-specific Protein Lysine Acetylation and Succinylation Stoichiometry Using Data-independent Acquisition Mass Spectrometry. *J Vis Exp.* 2018(134).
15. Schluter A, Real-Chicharro A, Gabaldon T, Sanchez-Jimenez F, and Pujol A. PeroxisomeDB 2.0: an integrative view of the global peroxisomal metabolome. *Nucleic acids research.* 2010;38(Database issue):D800-5.
16. Calvo SE, Clauser KR, and Mootha VK. MitoCarta2.0: an updated inventory of mammalian mitochondrial proteins. *Nucleic acids research.* 2016;44(D1):D1251-7.
17. MacLean B, Tomazela DM, Shulman N, Chambers M, Finney GL, Frewen B, et al. Skyline: an open source document editor for creating and analyzing targeted proteomics experiments. *Bioinformatics.* 2010;26(7):966-8.
18. Bharathi SS, Zhang Y, Gong Z, Muzumdar R, and Goetzman ES. Role of mitochondrial acyl-CoA dehydrogenases in the metabolism of dicarboxylic fatty acids. *Biochemical and biophysical research communications.* 2020;527(1):162-6.
19. Zhang Y, Bharathi SS, Beck ME, and Goetzman ES. The fatty acid oxidation enzyme long-chain acyl-CoA dehydrogenase can be a source of mitochondrial hydrogen peroxide. *Redox Biol.* 2019;26:101253.
20. Ackers-Johnson M, Li PY, Holmes AP, O'Brien SM, Pavlovic D, and Foo RS. A Simplified, Langendorff-Free Method for Concomitant Isolation of Viable Cardiac Myocytes and Nonmyocytes From the Adult Mouse Heart. *Circulation research.* 2016;119(8):909-20.
