# Supplementary material for: Dietary dicarboxylic acids provide a nonstorable alternative fat source that protects mice against obesity
Source: J Clin Invest. 2024 Apr 30;134(12):e174186. doi: 10.1172/JCI174186 (PMC11178532; doi:10.1172/JCI174186)

Unedited Blot Images

Goetzman et al

Blots from Fig 2b

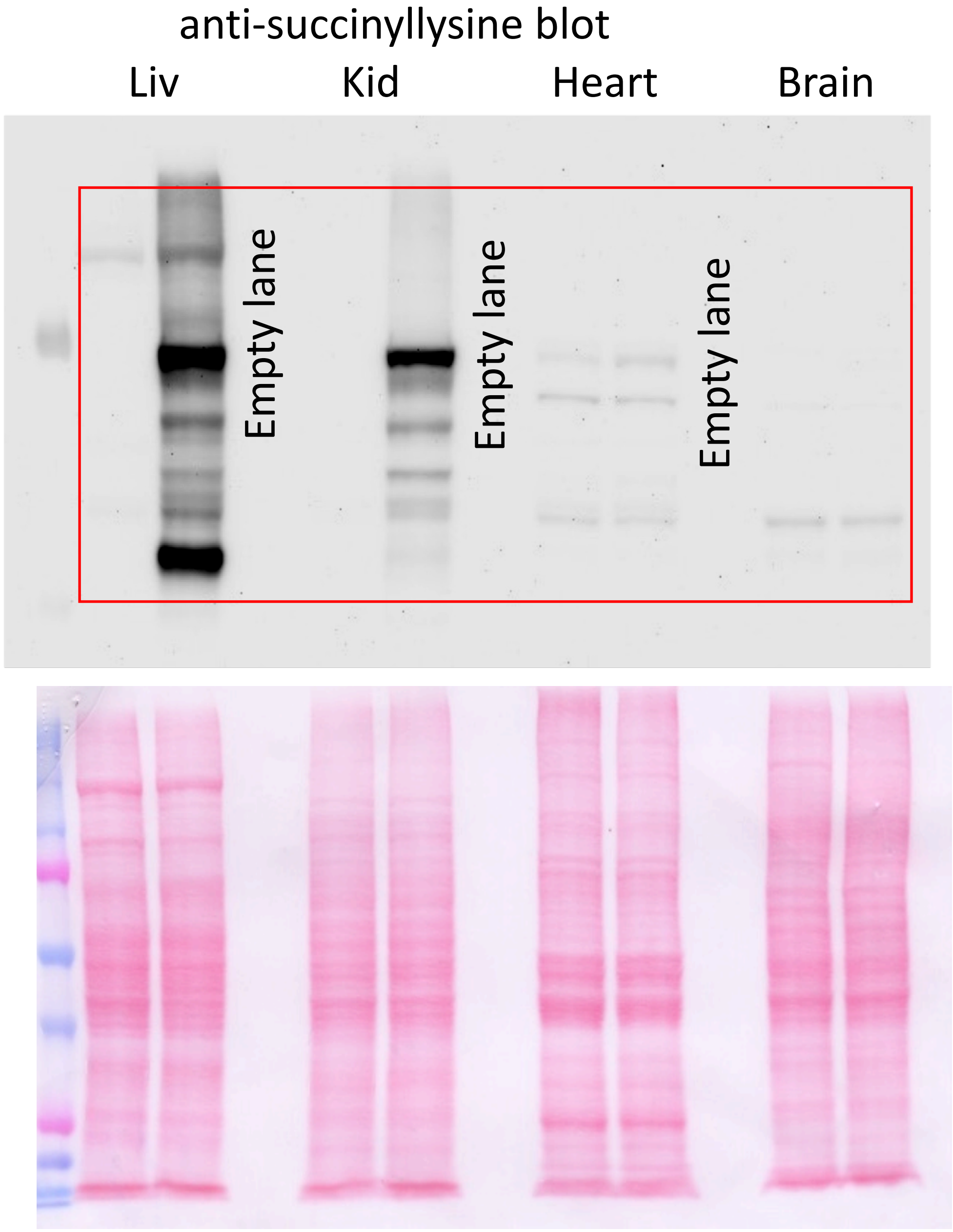

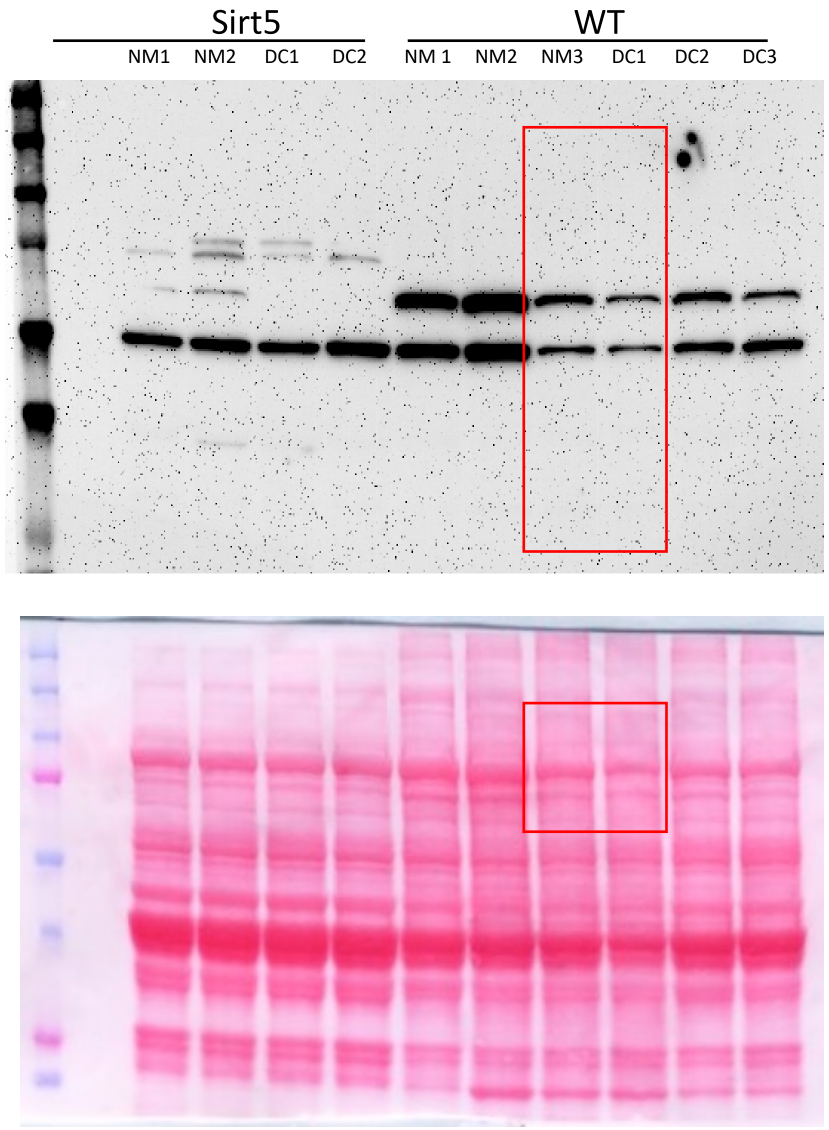

Fig 5e: iWAT UCP1-blot

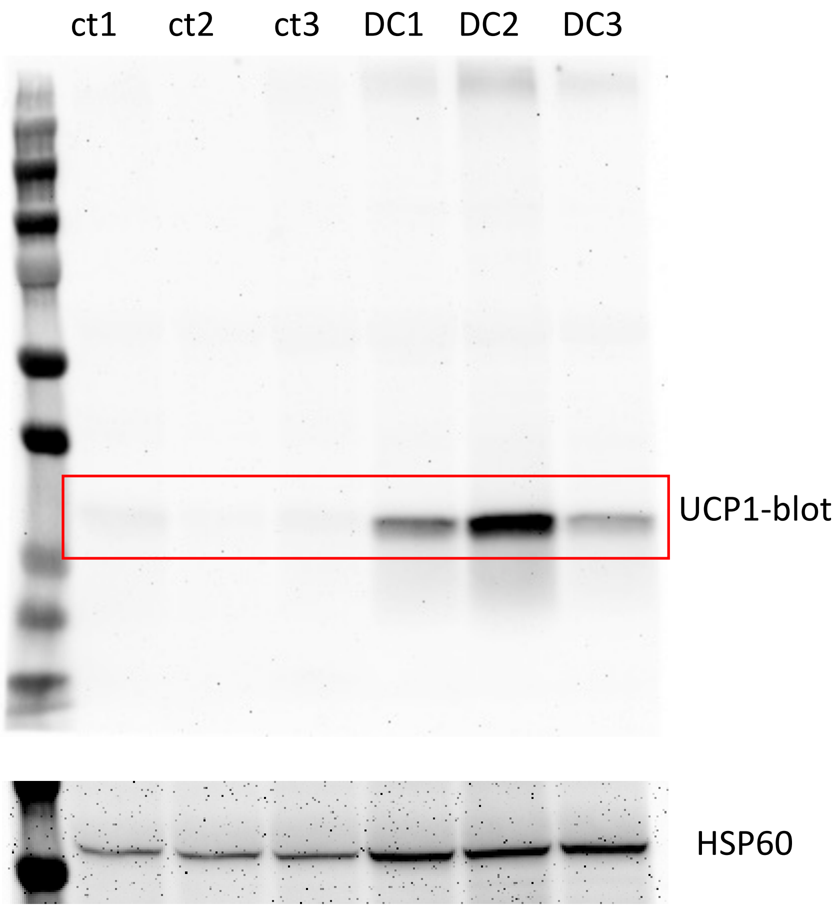

Supplemental Figure 4a

ACOX1

Note that ACOX1 has two bands. For simplicity, we showed only the top 72 kDa band in the manuscript.

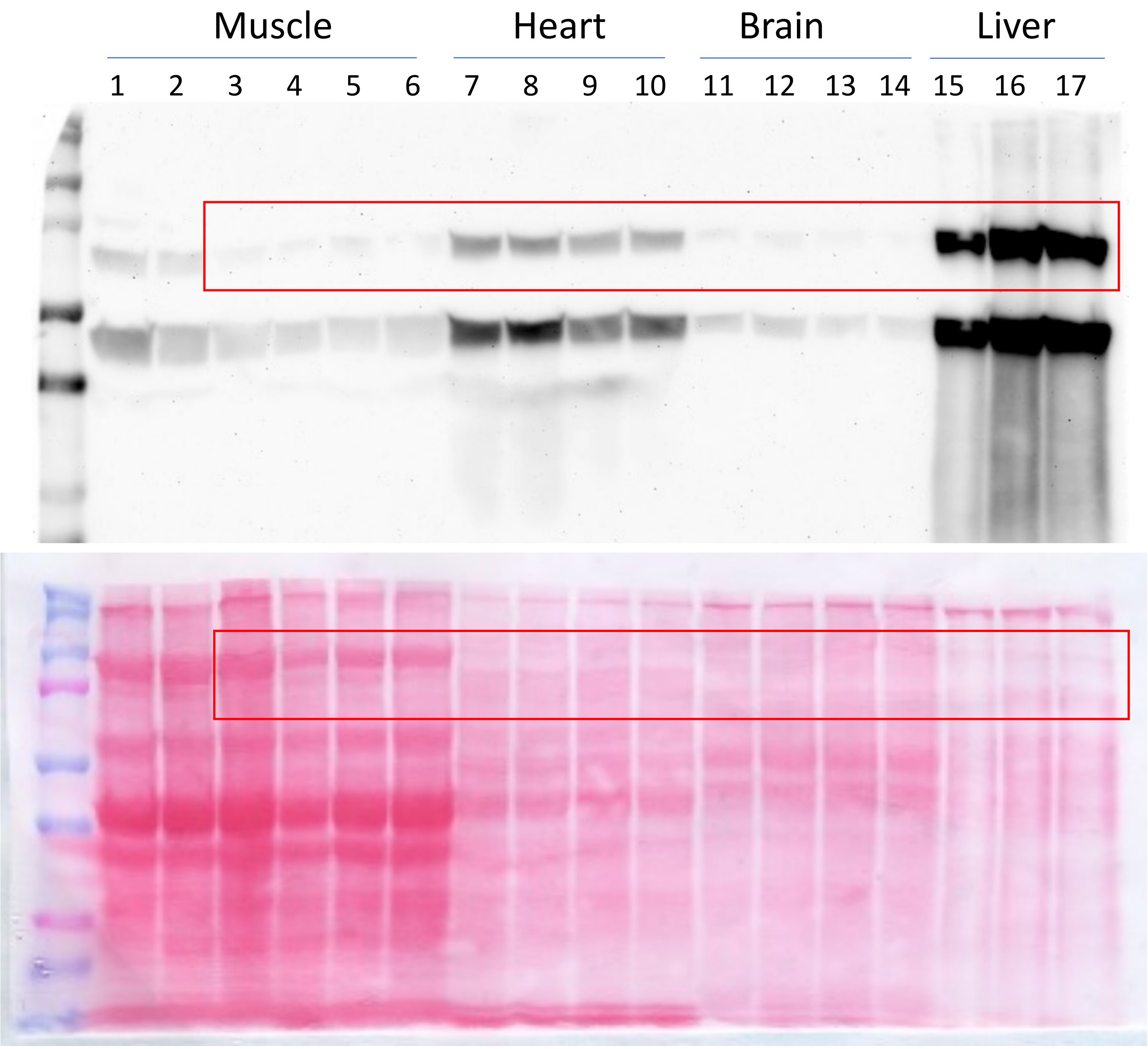

Ponceau

Muscle, heart, brain =45 ug
Liver = 15 ug

Supplemental Figure 4b

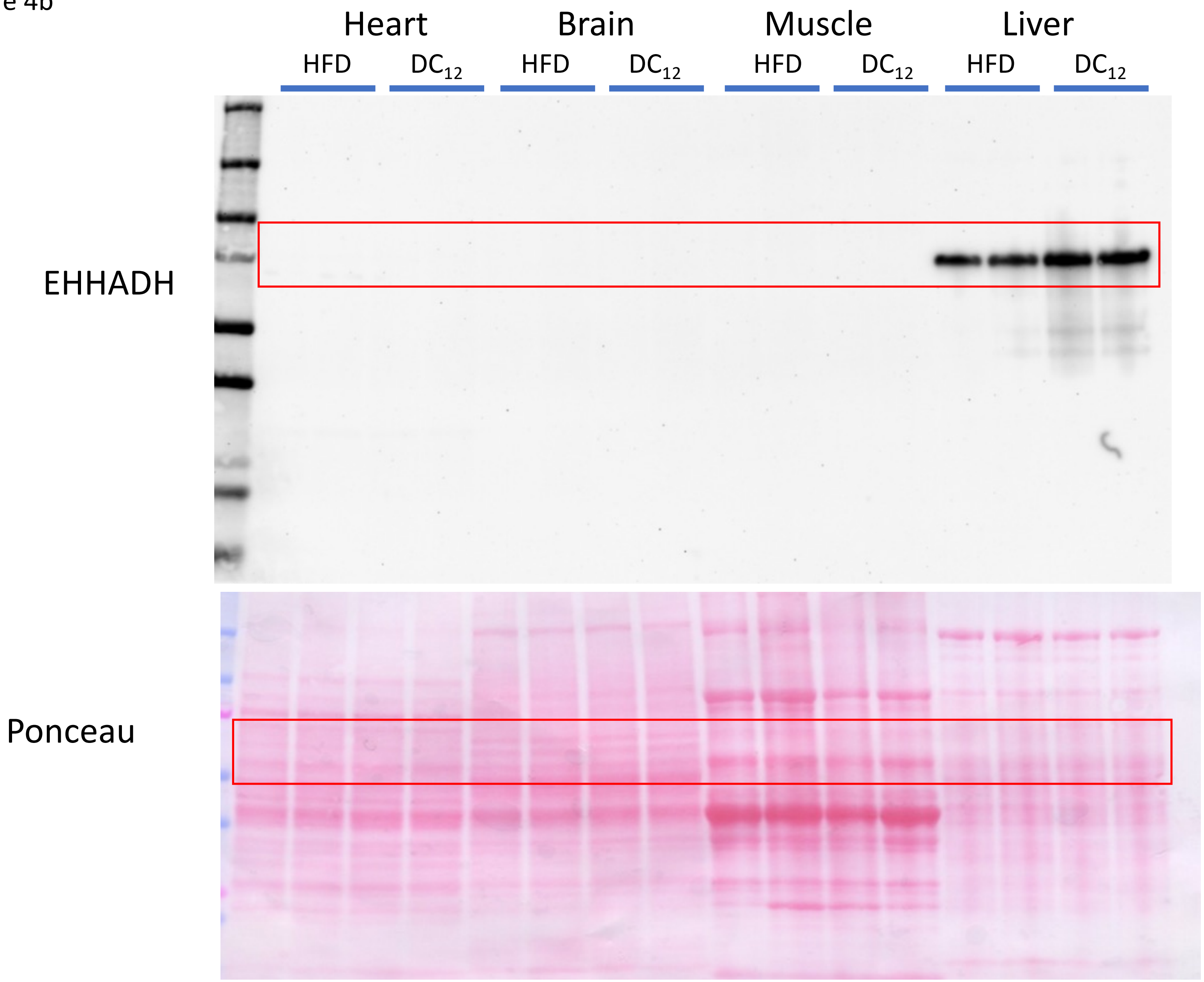

Supplement: Unedited blot and gel images [file jci-134-174186-s052.pdf]
